# Antiproliferative and Morphological Analysis Triggered by Drugs Contained in the Medicines for Malaria Venture COVID-Box Against *Toxoplasma gondii* Tachyzoites

**DOI:** 10.3390/microorganisms12122602

**Published:** 2024-12-16

**Authors:** Andréia Luiza Oliveira Costa, Mike dos Santos, Giulia Caroline Dantas-Vieira, Rosálida Estevam Nazar Lopes, Rossiane Claudia Vommaro, Érica S. Martins-Duarte

**Affiliations:** 1 Laboratório de Quimioterapia de Protozoários Egler Chiari, Departamento de Parasitologia—ICB, Universidade Federal de Minas Gerais, Belo Horizonte 31270-901, MG, Brazil; andreialuizaaa594@gmail.com (A.L.O.C.); mikesnts@outlook.com (M.d.S.); giuliacdv@outlook.com (G.C.D.-V.); rosalidaenlopes@gmail.com (R.E.N.L.); 2 Laboratório de Ultraestrutura Celular Hertha Meyer, Instituto de Biofísica Carlos Chagas Filho, Centro de Pesquisa em Medicina de Precisão, Universidade Federal do Rio de Janeiro, Rio de Janeiro 21941-902, RJ, Brazil; vommaro@biof.ufrj.br

**Keywords:** toxoplasmosis, drug repositioning, cycloheximide, bortezomib, midostaurin, (-)-anisomycin, almitrine

## Abstract

*Toxoplasma gondii* is a protozoan, and the etiologic agent of toxoplasmosis, a disease that causes high mortality in immunocompromised individuals and newborns. Despite the medical importance of toxoplasmosis, few drugs, which are associated with side effects and parasite resistance, are available for its treatment. Here, we show a screening of molecules present in COVID-Box to discover new hits with anti-*T. gondii* activity. COVID-Box contains 160 molecules with known or predicted activity against SARS-CoV-2. Our analysis selected 23 COVID-Box molecules that can inhibit the tachyzoite forms of the RH strain of *T. gondii* in vitro by more than 70% at 1 µM after seven days of treatment. The inhibitory curves showed that most of these molecules inhibited the proliferation of tachyzoites with IC_50_ values below 0.80 µM; Cycloheximide and (-)-anisomycin were the most active drugs, with IC_50_ values of 0.02 μM. Cell viability assays showed that the compounds are not toxic at active concentrations, and most are highly selective for parasites. Overall, all 23 compounds were selective, and for two of them (apilimod and midostaurin), this is the first report of activity against *T. gondii*. To better understand the effect of the drugs, we analyzed the effect of nine of them on the ultrastructure of *T. gondii* using transmission electron microscopy. After treatment with the selected drugs, the main changes observed in parasite morphology were the arrestment of cell division and organelle alterations.

## 1. Introduction

*Toxoplasma gondii* is the etiologic agent of toxoplasmosis, a zoonosis with a high proportion of seropositive individuals worldwide [1]. The importance of this disease for humans is related to the high morbidity of immunocompromised individuals, such as AIDS patients and newborns [2]. *T. gondii* infection is asymptomatic in 80% of infected individuals. However, primary infection in AIDS patients can cause the cerebral form of the disease [3].

Congenitally infected newborns can develop neurological problems and eye diseases [4]. In Europe, a risk of eye damage cases of 0.3% to 1% has been observed in adults one or two years after acquiring the infection, and retinochoroiditis results in higher damage in America than in Europe or North America [5,6]. It is believed that 400 to 4000 children are born with toxoplasmosis each year in the United States [7]. In Brazil, the incidence of congenital transmission can reach 1:770 live births [4], and the estimated prevalence of toxoplasmosis is 42 to 92%, depending on the region of the country [8].

Despite the medical importance of toxoplasmosis, few drugs are available for its treatment [9]. The combination of pyrimethamine (PYR), sulfadiazine (SDZ), and folinic acid has been administered for approximately 70 years. This association is still the first choice for all clinical conditions of the disease [10]. However, this therapeutic scheme has some critical flaws, such as frequent and non-tolerated side effects, which lead to the abandonment of treatment. In addition, there are reports of therapeutic failures with *T. gondii* strains possibly resistant to these drugs, as well as low absorption and the need for high therapeutic doses or a long-period treatment, making it challenging to manage the treatment [11,12,13].

In this context, drug repositioning has been a promising strategy for new treatments for infectious and neglected diseases. This strategy consists of redirecting an active pharmaceutical compound with commercial use or in development and reusing it for a new therapeutic indication. The development and discovery of medicines are long and high-investment processes [14,15]. Based on this, the Medicines for Malaria Venture (MMV) develops and offers several promising drug libraries with potential repositioning for treating neglected diseases and infections that can cause epidemics. The first drug box provided by MMV was in 2014, Malaria-Box. The anti-*T. gondii* activity of the compounds available in Malaria-Box has already been described in the literature [16,17,18]. Several compounds in other MMV boxes, such as Pathogen and Pandemic, have also shown anti-*T. gondii* activity [19,20,21,22].

In 2020, with the advent of the COVID-19 pandemic, MMV made available a new box with 160 drugs and compounds, COVID-Box. The drugs and compounds in this box have structurally different therapeutic classes, selected by experts and initially tested against the new coronavirus (SARS-CoV-2). As COVID-Box compounds are in various stages of pharmacological research and development, it is interesting to identify new potential treatments for toxoplasmosis. Previous work demonstrated the activity of those compounds in *T. gondii*, confirming the potential of COVID-Box [23,24]. Thus, in this work, we aimed to expand the study of the activity of those compounds against the tachyzoites of *T. gondii* and characterize the mode of action of the best ones by morphological analysis using fluorescence and transmission electron microscopy.

## 2. Materials and Methods

### 2.1. Drugs and Compounds of COVID-Box

The 160 drugs and compounds were provided free of charge by MMV: https://www.mmv.org/mmv-open/covid-box/covid-box-supporting-information (accessed on 12 August 2024). The compounds were provided solubilized in DMSO at 10 mM in two 96-well plates (A and B) containing 80 compounds each. For storage in this box, the drugs and compounds were dissolved in reserves of 2 mM with 100% DMSO (Merck, Darmstadt, Germany).

### 2.2. Parasites

*Toxoplasma gondii* tachyzoites of the RH strain were used. The parasites were maintained in vitro through serial passages in 25 cm^2^ culture flasks containing confluent neonatal normal human dermal fibroblastic cells (NHDF; Lonza, kindly donated by Dr. Sheila Nardelli, Fiocruz Paraná) in RPMI 1640 (Gibco) medium supplemented with 2% fetal bovine serum (Gibco), penicillin/streptomycin, amphotericin B (Life Technologies, Eugene, OR, USA), and 2 mM glutamine (complete medium). Cells and parasites were maintained at 37 °C in a humid atmosphere of 5% CO_2_.

### 2.3. Antiproliferative Assays Against Tachyzoite Stage

For assessing the proliferation of tachyzoites, we used the classical plaque assay. For preliminary evaluation of the 160 drugs and compounds in the COVID-Box, 6-well plates with monolayers of NHDF cells in complete RPMI-1640 medium were infected with 1000 newly egressed tachyzoites of *T. gondii* and treated with 1 µM of each drug. For antiproliferative curves, 12-well plates were used, and cells were infected with 600 tachyzoites. After adding the parasites, decreasing concentrations of drugs and compounds (1–0.0078 µM in two-fold serial dilutions) were added to each well. In the control wells, 0.1% DMSO was added. During the treatment period, the plates were maintained stable and without disturbing to not spread the egressed parasite from the infection focus (plaques). After 7 days of treatment, the cells were fixed with 70% ethanol and stained with crystal violet. Stained plates were imaged with ChemiDoc MP Imaging System (Bio-Rad, Hercules, CA, USA), and then plaque areas were analyzed with the ImageJ^®^ software (version 1.52e). The destruction areas of treated cells were quantified and compared with the untreated (control) to determine the percentage of proliferation and inhibition of *T. gondii*. For the calculation of the inhibitory concentration of 50% (IC_50_), the growth inhibition percentage was plotted as a function of drug concentration by fitting the values to the standard curve analysis. The regression analyses were performed using GraphPad Prism 8 Software (GraphPad Inc., San Diego, CA, USA).

### 2.4. Cytotoxicity Assay in NHDF Cell

The cytotoxic effect of COVID-Box drugs and compounds against NHDF cells was evaluated by the MTS assay [25,26]. For this, 96-well tissue plates containing NHDF cells were treated with different concentrations of drugs and compounds for seven days. At the end of the treatment, the cells were washed with phosphate-buffered saline (PBS, pH 7.4), each well was filled with 100 μL of 10 mM glucose in PBS, and 20 µL of MTS reagent (Promega, Madison, WI, USA) was added. The absorbance was read at 490 nm after three hours of incubation at 37 °C. Cytotoxicity was calculated as the percentage of viable cells versus untreated cells (control). The cytotoxic concentration of 50% (CC_50_) for the host cells was calculated as for IC_50_. The selective index (SI) was calculated as the ratio of CC_50_/IC_50_.

### 2.5. Druggability Analysis of Drugs and Compounds

For the in silico analysis, the chemical structures of the simplified molecular line input system (SMILES) code from all drugs were obtained from the plate map available at https://www.mmv.org/mmv-open/covid-box/covid-box-supporting-information and loaded into the online programs pkCSM (https://biosig.lab.uq.edu.au/pkcsm/prediction) and SwissADME (http://www.swissadme.ch/) for physicochemical, toxicity and pharmacokinetics properties analysis of the drugs and compounds of COVID-Box. Pyrimethamine (PYR), sulfadiazine (SDZ), clindamycin (CLI), azithromycin (AZT), and atovaquone (ATO) were analyzed as reference drugs.

### 2.6. Twenty-Four Hours Antiproliferative Assay

To validate the antiproliferative effect of the compounds with the highest activity present in COVID-Box, 24-well plates containing NDHF cell monolayer coverslips were infected at a 5:1 ratio (tachyzoite/cell) with fresh infusions of the RH strain for two hours. The cells were then washed twice with PBS pH 7.4 to remove non-invaded parasites and incubated for another two hours with fresh medium. At the end of this time, the cells were treated with 1 µM almitrine, 62.5–125 nM (-)-anisomycin, 0.5–1 µM apilimod, 31.2–62.5 nM bortezomib, 31.2–125 nM cycloheximide, 1 µM ivermectin, 1.5 and 2.0 µM merimepodib, 0.25–1 µM midostaurin, 125–250 nM mycophenolic acid, and 31.2–250 nM salinomycin for 24 h. At the end of this time, the washing, fixation, and cell counting steps were followed as described above [21]. The proliferation index is the product of the total number of tachyzoites and the total number of cells divided by the percentage of infected cells [27].

### 2.7. Transmission Electron Microscopy (TEM)

NHDF cultures infected with *T. gondii* were treated with compounds and fixed with 2.5% glutaraldehyde (EM Grade—Ted Pella Inc., Redding, CA, USA) in 0.1 M sodium cacodylate buffer pH 7.4 (Ted Pella Inc.). Cells were post-fixed with 1% osmium tetroxide, 1.25% potassium ferrocyanide, and 5 mM CaCl_2_, in 0.1 M sodium cacodylate buffer (pH 7.4). The sample fixation and processing for microscopy were performed as previously described [28]. Samples were dehydrated in alcohol solutions of increasing concentrations (35–100%) and embedded in epoxy resin (Polybed 812, Polysciences). Ultrathin sections were collected in copper grids 300 mesh (Ted Pella Inc.), stained with uranyl acetate and lead citrate, and then observed in a Fei TecNai G2 120 kV Spirit Electron Microscope (FEI Company, Eindhoven, The Netherlands).

### 2.8. Immunofluorescence Microscopy

For the immunofluorescence microscopy, NHDF cells were infected with newly egressed tachyzoites of *T. gondii* in the ratio 5:1 and treated for 24 h with 1 µM ivermectin, 0.25–0.5 nM midostaurin, 125 nM salinomycin, 1.5 µM merimepodib, 62.5–125 nM (-)-anisomycin, 31.2–62.5 nM bortezomib, and 1 µM almitrine. When completing the treatment, the cells were prepared as previously described [28]. Rabbit anti-IMC1 (1:1000) and anti-ARO (1:1000) (kindly provided by Dr. Dominique Soldati, Universite de Geneve, Switzerland) were used to label the mother cell and daughter cell pellicles throughout the endodyogeny process and rhoptries, respectively. Mouse anti-SAG1 (1:200; kindly donated by Dr. Tiago Mineo, Universidade Federal de Uberlândia) was used to label the parasite plasma membrane. DAPI (2 µg/mL; Sigma-Aldrich, St. Louis, MO, USA) was used to label the DNA. Goat anti-rabbit IgG conjugated to Alexa-488 and goat anti-mouse IgG conjugated to Alexa 546 were used as secondary antibodies (Life Technologies, Eugene, OR, USA). After labeling with antibodies, the coverslips were mounted onto slides using Prolong gold (Life Technologies), and samples were examined on a Zeiss Axio Observer Z1 microscope (Baden-Württemberg, Germany) or Olympus BX60 microscope (Shinjuku City, Tokyo, Japan).

### 2.9. Statistical Analysis

Data were analyzed using GraphPad Prism 8.0 software (GraphPad Inc., San Diego, CA, USA). IC_50_ and CC_50_ calculations were performed by fitting the values of proliferation/viability in percentage to a non-linear curve followed by dose-response inhibition analysis through log(inhibitor) vs. normalized response. One-way ANOVA and *t*-test were used for the quantitative analysis.

## 3. Results

### 3.1. Drugs of COVID-Box Showed High Activity and Selectivity Against T. gondii Tachyzoites

The antiproliferative effect of 1 µM of each of the 160 drugs and compounds present in the COVID-Box (Supplementary Table S1) was screened against tachyzoites of the RH strain of *T. gondii* for seven days of treatment. Thirty compounds inhibited parasite proliferation by at least 70% (Supplementary Figure S1). Of the thirty best drugs, seven were excluded from the studies. Two (amiodarone and proscillaridin) were initially excluded because they presented signs of cytotoxicity for the host cells during the initial screening. Five other drugs (itraconazole, doxycycline, cyclosporine, doxorubicin, and digitoxin) were excluded as their activity against *T. gondii* has been extensively studied before [24,29,30,31,32,33,34]. Details of the plaque assay of the best selected 23 compounds and drugs are in the Supplementary Figure S2. The chosen drugs (Supplementary Figure S3) were studied for the IC_50_ determination and cytotoxicity analysis (Supplementary Figures S4 and S5 and Table 1).

The drugs Cycloheximide, Bortezomib, Digoxin, and (-)-Anisomycin were the most active, inhibiting the proliferation of *T. gondii* with IC_50_s values lower than or equal to 30 nM (Table 1). Salinomycin, mycophenolic acid, abemaciclib, midostaurin, emetine, and LY2228820 inhibited *T. gondii* proliferation with IC_50_ lower than 100 nM (Table 1). The drugs ivermectin, almitrine, apilimod, bemcentinib, niclosamide, regorafenib, and merimepodib were also highly active against *T. gondii*, presenting IC_50_ in the range of 0.15–0.48 µM (Table 1).

The cytotoxicity assay showed that most compounds were highly selective against *T. gondii*, and the SI ranged from 3 to 304 (Table 1). Drugs with IC_50_s less than 30 nM (cycloheximide and bortezomib) had the highest SI. Overall, all 23 compounds were selective, and for two of them (apilimod and midostaurin), this is the first report of activity against *T. gondii* (Table 1).

### 3.2. COVID-Box Drugs Show Potential Oral Druggability

Through the SwissADME [44] platform, it was possible to obtain information about the physical-chemical properties of the drugs that showed the best activity against *T. gondii*. From these analyses, it was possible to predict whether these compounds are by the predictors of Lipinski’s rule of five (RO5) and Veber (Table 2). We also compared PYR, SDZ, CLI, AZT, and ATO, which are currently used for treating toxoplasmosis. The RO5 states that drugs with more than 5H-bond donors, more than 10H-bond acceptors, a molecular weight (MW) greater than 500, and a calculated LogP (a measure of lipophilicity) greater than five are less likely to have good oral absorption and permeation. In addition to the RO5 of Lipinski et al. (1997) [45], the two predictors of Veber et al. (2002) [46] also indicate that compounds with total polar surface area (TPSA) equal to or <140 Å^2^ and with ten or less rotating bonds have a greater chance of success in oral bioavailability.

The analyses were carried out for the 23 drugs from the COVID-Box selected in the antiproliferative assay and those used as the gold standard (PYR and SDZ) and alternatives (AZT, CLI, and ATO) for toxoplasmosis. As expected, PYR and SDZ results agreed with Lipinski’s RO5 and Veber’s predictors. Among the drugs used as alternative treatments, only AZT violates two of Lipinski’s rules (MW and 5H-bond donors) and one Veber predictor (TPSA < 140 Å^2^) (Table 2). Of the 23 selected from the COVID-Box, 10 (niclosamide, apilimod, regorafenib, emetine, sorafenib, mycophenolic acid, merimepodib, cycloheximide, (-)-anisomycin, and bortezomib) showed compliance with the RO5 and Veber’s predictors (Table 2). The other drugs and compounds showed at least one or more non-compliances with the RO5 and Veber (Table 2).

Information on the pharmacokinetic properties of selected drugs from the COVID-Box and drugs already used in treating toxoplasmosis was obtained using the platform pkCSM (Supplementary Table S2). Caco-2 permeability values (log Papp at 10^−6^ cm/s) above 0.90 predict high intestinal permeability. The drugs bemcentinib, apilimod, bortezomib, manidipine, almitrine, midostaurin, abemaciclib, and ponatinib had a log Papp at 10^−6^ cm/s above 0.90, and regorafenib, emetine, sorafenib, tetrandrine, and mycophenolic acid showed values of log Papp at 10^−6^ cm/s > 0.70– < 0.90, from which it can be inferred that these also have the potential to present high intestinal permeability [47]. The other drugs showed values below 0.60 log Papp at 10^−6^ cm/s (Table S2). It should also be noted that even the compound cycloheximide, presenting a Caco-2 value below 0.90 (0.553 log Papp at 10^−6^ cm/s, intestinal absorption (human) = 69.8%) (Table S2) was the compound selected in the in vitro tests that most inhibited parasite proliferation with an IC_50_ value = 0.02 μM (Table 1).

The volume of distribution value (VDss) predicts the drug distribution in tissue. It is known that the lower the interaction of drugs with plasma proteins, the faster they will be absorbed, and, therefore, the faster they will be directed to their site of action. Thus, the higher the VDss value above 0.45 log L/kg, the more the drug is distributed in tissues than in plasma, and values below −0.15 log L/kg are considered poorly distributed [47]. Seven drugs or compounds selected from the COVID-Box (bemcentinib, emetine, ivermectin, manidipine, almitrine, abemaciclib, and ponatinib) showed high distribution. Two drugs used for the treatment of toxoplasmosis (SDZ and atovaquone) and 0.329), and the COVID-Box drugs niclosamide, LY2228820, digoxin, sorafenib, salinomycin, merimepodib, cycloheximide, (-)-anisomycin, and pimozide showed VDss between −0.15 and 0.45 log L/kg (Table S2). Fraction unbound analyses showed that (-)-anisomycin and cycloheximide were the drugs with a higher proportion of free state in the plasma (Table S2).

The central nervous system (CNS) is a common site of infection of *T. gondii*; thus, we evaluated the predictors for CNS and BBB permeability of the COVID-Box drugs (Table S2). For CNS permeability, compounds with logPS > −2 are predicted to penetrate but with logPS < −3 are unable to penetrate. From the COVID-Box, six drugs (niclosamide, LY2228820, sorafenib, midostaurin, rapamycin, and pimozide) showed a prediction of CNS penetration, and twelve drugs presented predicted logPS between −3 and −2 and have the potential for penetration too (Table S2).

For BBB permeability, LogBB values above 0.3 predict that a compound could readily cross the BBB, and the ones with <−1 are poorly permeable [47]. None of the drugs and compounds selected from COVID-Box and most of the current ones used for toxoplasmosis treatment showed prediction for a high crossing into the brain. However, thirteen (niclosamide, bemcentinib, emetine, manidipine, almitrine, midostaurin, tetrandrine, ponatinib, berbamine, mycophenolic acid, cycloheximide, (-)-anisomycin, and pimozide) showed values > −1. Of the drugs already used in the treatment of toxoplasmosis, the only one with a value above 0.3 was ATO (0.401 log BB). PYR presented a value close to the expected value (0.278 log BB) (Table S2).

Using the SwissADME platform, we obtained the boiled-Egg graph, which also predicts if the drugs have the potential to cross the BBB and have high gastrointestinal absorption (HIA). The BBB permeability data provided by pkCSM were compared with the data provided in the Swiss ADME boiled-Egg plot [44]. Of the analyzed drugs used for toxoplasmosis treatment, only PYR and ATO showed characteristics with potential BBB permeability at the points drawn above the egg yolk in the graph (yellow color) (Supplementary Figure S6). The pkCSM program predicted that only ATO could cross the BBB (Supplementary Table S2). On the same graphic, those that are plotted in the egg white region (SDZ and CLI) would be more easily absorbed in the gastrointestinal tract by passive transport than the compounds that were plotted in the gray area of the graph (AZT) (Figure S6A). In addition, the graph provides information such as whether the drugs are glycoprotein inhibitors. Only PYR, SDZ, and ATO drugs are not P-gp substrates (marked with red dots in the graph) (Figure S6A). Compounds that are P-gp inhibitors show increased absorption, while P-gp substrates reduce their absorption [48].

Of COVID-Box, almitrine (Figure S6B), emetine, and ponatinib (Figure S6C) presented characteristics with potential permeability through the BBB (points drawn in the upper part of the yolk of the graph) (Figure S6B,C); this information complies with the results obtained for BBB permeability presented in Table S2. According to the results obtained in this analysis, none of the others are predictable for readily crossing the natural protections of the CNS [49]. The drugs niclosamide, bemcentinib, apilimod, LY 2228820, digoxin, manidipine, midostaurin, abemaciclib, tetrandrine, berbamine, mycophenolic acid, merimepodib, cycloheximide, (-)-anisomycin, bortezomib, and pimozide (Figure S6B,C) show potential for easier absorption in the gastrointestinal tract by passive transport. Of the COVID-Box drugs, niclosamide, apilimod, regorafenib, sorafenib, almitrine, tetrandrine, berbamine, mycophenolic acid, salinomycin, cycloheximide, and (-)-anisomycin are predicted as non-P-gp substrates (marked with red dots in the graph) (Figure S6B). Bemcentinib, LY 2228820, digoxin, emetine, ivermectin, manidipine, abemaciclib, ponatinib, merimepodib, bortezomib, and pimozide are P-gp substrates (marked with blue dots in the graph Figure S6C).

In agreement with the ADME in silico analysis, pharmacokinetics in in vivo studies showed that the selected drugs are absorbed and available in the plasma (Table S3). Brain availability was confirmed for abemaciclib, (-)-anisomycin, apilimod, bemcentinib, emetine, ivermectin, ponatinib, regorafenib, salinomycin, sorafenib, and tetrandrine (Table S3).

### 3.3. Twenty-Four-Hour Antiproliferative Assay

**Figure 1:**
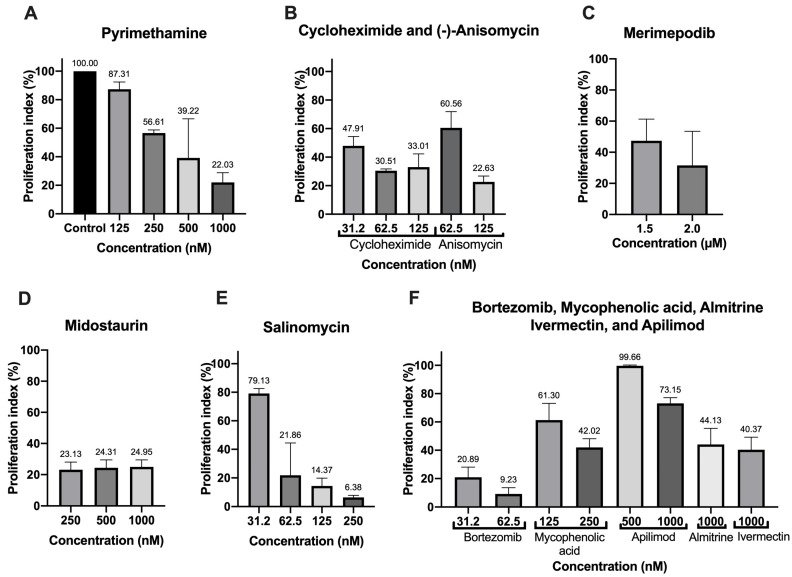
Proliferation index of the best drugs of the COVID-Box after 24 h of treatment with different concentrations of (**A**) Pyrimethamine, (**B**) Cycloheximide, Anisomycin, (**C**) Merimepodib, (**D**) Midostaurin, (**E**) Salimomycin, (**F**) Bortezomib, Mycophenolic acid, Apilimod, Almitrine, and Ivermectin. Values represent mean ± SD of three experiments, except for merimepodib, salinomycin, and pyrimethamine (two experiments).

For the drugs with the best IC_50_ value (cycloheximide, bortezomib, (-)-anisomycin, mycophenolic acid, and salinomycin), good predictable druggability in the in silico analysis (almitrine and merimepodib), or first reported for anti-*T. gondii* activity (apilimod and midostaurin), we carried out a proliferation assay to verify the inhibitory capacity of the compounds after only 24 h of treatment (Figure 1). As a positive control, we used PYR (Figure 1A). The treatment with 125 nM (-)-anisomycin (Figure 1B), 31.2 nM bortezomib (Figure 1F), 0.25 µM midostaurin (Figure 1D), and 62.5 nM cycloheximide (Figure 1B) inhibited the proliferation around 75–80%. The treatment with 250 nM salinomycin and 62.5 nM bortezomib reduced the proliferation of the parasite by more than 90% (Figure 1E,F). Treatment with 1.5 and 2.0 µM merimepodib (Figure 1C), 1000 nM almitrine, 250 nM mycophenolic acid, and 1000 nM ivermectin (Figure 1E) inhibited the parasite proliferation around 55–60%. Apilimod only showed a modest inhibition after 24 h of treatment (Figure 1E).

### 3.4. Analysis of the Effect of the Drugs and Compounds of COVID-Box on the Parasite Morphology by Transmission Electron Microscopy (TEM) and Fluorescence Microscopy

**Figure 2:**
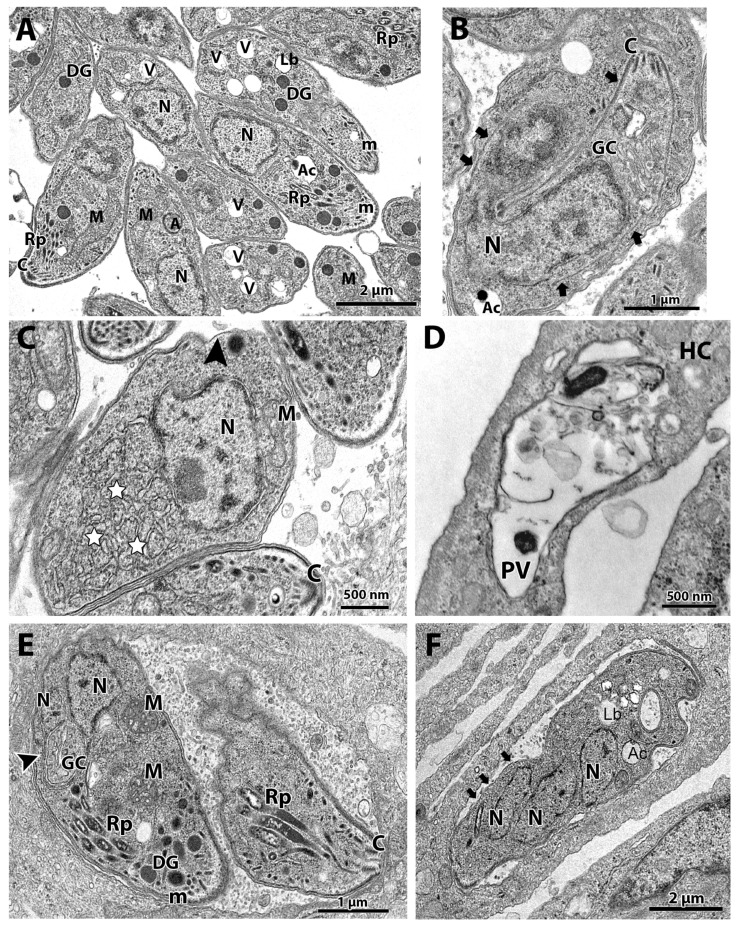
(**A**–**F**). Transmission electron microscopy and analysis of the ultrastructure of tachyzoites after treatment with cycloheximide and bortezomib. The parasites were treated with the compounds for 48 h. (**A**,**B**) Untreated parasites showed typical morphology (**A**,**B**) and division process by endodyogeny (arrows in (**B**)). (**C**) Parasites treated with the drug 62.5 nM cycloheximide showed an increase in the endoplasmic reticulum area (stars) and alterations in the structure of the plasma membrane, with regions with a lack of inner membrane complex (black arrowhead). (**D**) Parasites treated with 125 nM cycloheximide were destroyed; it is possible to observe parasite content spread through the PV. (**E**,**F**) Parasites treated with 62.5 nM bortezomib showed cell division alterations, as seen by the Golgi complex surrounded by the nucleus envelope (arrowhead) and a parasite presenting three nucleus profiles without constructing new daughter cells. Mitochondrial swelling (M) and regions of parasite devoid IMC were also observed (arrows). A—apicoplast, Ac—acidocalcisome, C—conoid; DG—dense granules, GC—Golgi complex, Lb—lipid body, M—mitochondrion, m—micronemes, N—nucleus, Rp—rhoptries, PV—parasitophorous vacuole, V—vacuolar compartment.

**Figure 3:**
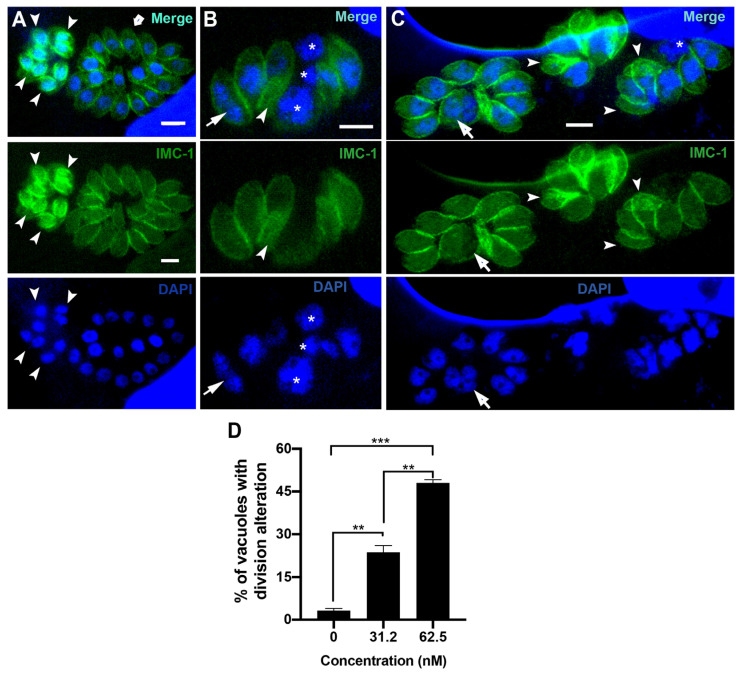
Fluorescence microscopy of untreated parasites (**A**) or after treatment with 31.2 nM (**B**) and 62.5 nM (**C**) of bortezomib. Parasites were labeled with anti-IMC1 for inner membrane complex (IMC, green) and DAPI for DNA (blue). (**A**) Untreated parasites showed typical morphology (arrow) and division process (arrowhead). (**B**,**C**) treated parasites showed an aberrant cell division process with large parasites harboring two or more nuclei (arrow), daughter cells without nuclei (arrowheads), and regions of the cells without IMC coverage (asterisks). (**D**) Quantitative analysis of the number of PVs presenting parasites with aberrant cell division. Results in (**D**) are the mean ± SD of two independent experiments. * *p* < 0.05; **p < 0.01; *** *p* < 0.001. Bars = 2.5 µm.

**Figure 4:**
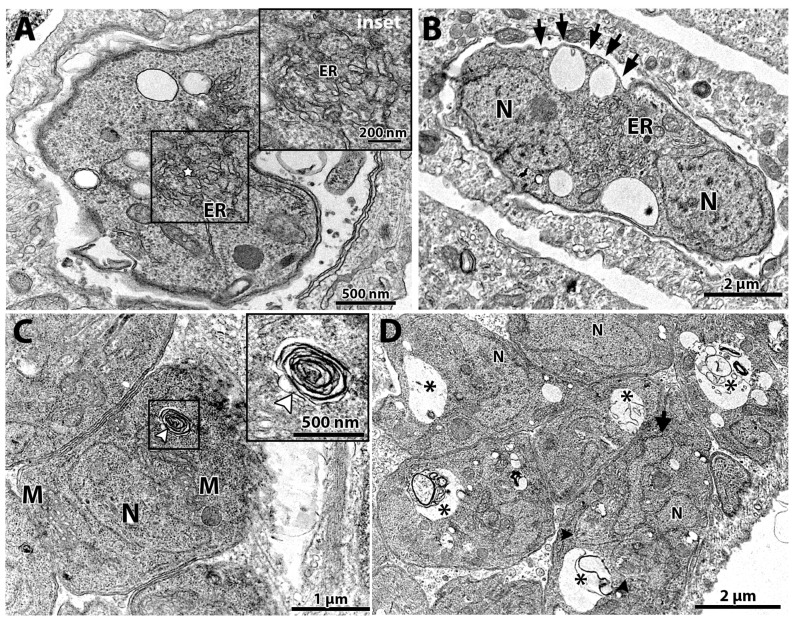
Transmission electron microscopy of *T. gondii* after treatment for 48 h with (-)-anisomycin (**A**,**B**) and ivermectin (**C**,**D**). (**A**) Treatment with 100 nM (-)-anisomycin induced changes in the parasite’s endoplasmic reticulum (star in inset) and (**B**) 100 nM (-)-anisomycin also induced impairment of the cell division, making it possible to observe a single parasite with two nuclei and causing discontinuation of the inner membrane complex (black arrows). (**C**) Parasites treated with 1 µM ivermectin induced the formation of myelin-like figures (inset—white arrowhead). (**D**) In this figure, it is also possible to observe an intense vacuolization process in parasites treated with 1 μM ivermectin (asterisks). M—mitochondria; N—nucleus; GC—Golgi complex; ER—endoplasmic reticulum.

**Figure 5:**
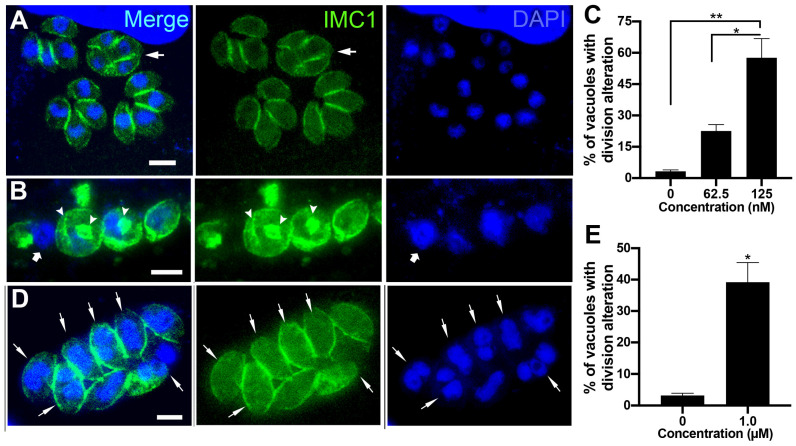
Fluorescence microscopy analysis of tachyzoites treated with 62.5 nM and 125 nM (-)-anisomycin (**A**–**C**) and 1 µM ivermectin (**D**,**E**). Parasites were labeled with anti-IMC1 for inner membrane complex (IMC, green) and DAPI for DNA (blue). (**A**) Parasites treated with 62.5 nM (-)-anisomycin showed daughter cells’ budding arrestment, forming a large mass of tethered daughter cells (arrow). (**B**) Treatment with 125 nM (-)-anisomycin led to a large round mass of cells with a nucleus of increased size and disorganized profiles of IMC (arrowheads). The arrow points to a parasite region without the IMC coverage. (**C**) Quantitative analysis of the number of PVs presenting parasites with aberrant cell division after treatment with (-)-anisomycin. (**D**) Parasites treated with 1 µM ivermectin showed a divided nucleus without the construction of daughter cells (arrows). (**E**) Quantitative analysis of the number of PVs presenting parasites with aberrant cell division after treatment with ivermectin. Results in (**C**,**E**) are the mean ± SD of two independent experiments. * *p* < 0.05; ** *p* < 0.01. Bars = 2 µm.

**Figure 6:**
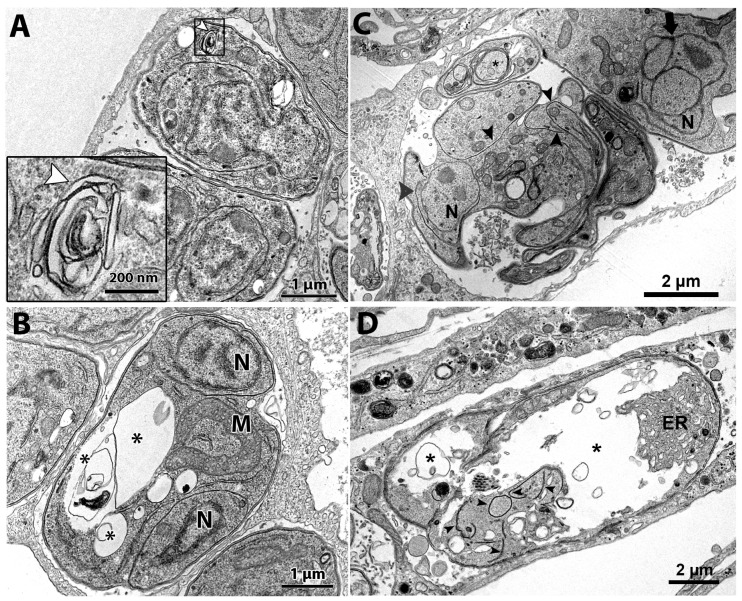
Transmission electron microscopy of *T. gondii* tachyzoites after treatment with almitrine and midostaurin for 48 h. (**A**) Parasites treated with 1 µM almitrine showed myelin-like structures (arrowhead in inset). (**B**) Treatment with one µM almitrine also induced the formation of large vacuoles containing membranous material (asterisks) and disruption of cell division, as seen by a large mother mass harboring two non-budded daughter cells (asterisks). (**C**,**D**) Parasites treated with 250 nM midostaurin for 48 h. (**C**) Vacuole containing a mass of tachyzoite with several arrested daughter cells and IMC profiles through the cytoplasm (arrowheads). A parasite presenting a fragmented nucleus (large arrow), and a process similar to autophagy (asterisks) was observed too.

**Figure 7:**
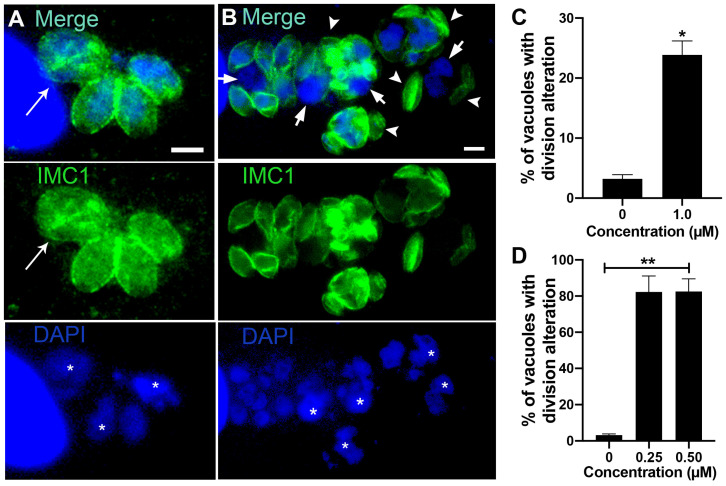
Fluorescence microscopy analysis of tachyzoites treated with almitrine and midostaurin. Parasites were labeled with anti-IMC1 for inner membrane complex (IMC, green) and DAPI for DNA (blue). (**A**) Parasites treated with 1 µM almitrine showed cell division alteration with tachyzoites presenting large nuclei (asterisks) and masses with incomplete division process (arrow). (**B**) Treatment with 0.25 µM midostaurine caused a large round mass of cells with a nucleus of increased size (asterisk), tachyzoites showing regions without the IMC cover (arrows), and daughter cells without a nucleus (arrowheads). (**C**) Quantitative analysis of the number of PVs presenting parasites with aberrant cell division after treatment with almitrine. (**D**) Quantitative analysis of the number of PVs presenting aberrant parasites after treatment with midostaurin. Results in (**C**,**D**) are the mean ± SD of two independent experiments. * *p* < 0.05; ** *p* < 0.01. Bars = 2.5 μm.

**Figure 8:**
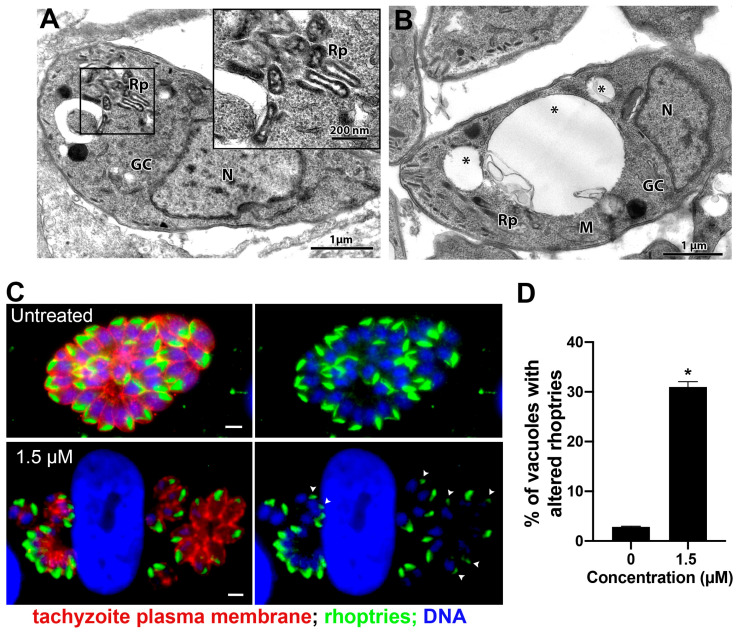
Morphological analysis of tachyzoites of *T. gondii* after treatment with 1.5 µM merimepodib. (**A**) Treatment with 1.5 µM induced Golgi complex fragmentation (vesiculation) and rhoptry disorganization, which can be seen at higher magnification in the inset. (**B**) Tachyzoites treated with 1.5 µM merimepodib also presented large vacuoles containing membranous material (asterisks). (**C**) Fluorescence microscopy analysis of tachyzoites treated with 1.5 µM merimepodib for 24 h. Parasites were labeled with anti-ARO for rhoptries (green), anti-SAG1 for parasite plasma membrane (red), and DAPI for DNA (blue). Images represent the projection of different Z focal planes. (**D**) Quantitative analysis of the number of PVs presenting parasites with rhoptry- altered morphology (arrowheads in (**C**)). Results are the mean ± SD of two independent experiments. * *p* < 0.05. M—mitochondria; N—nucleus; GC—Golgi complex; Rp—rhoptries. Bars = 2 µm.

To verify the direct effect and to determine the mode of action on *T. gondii* of the drugs, the effect on the ultrastructure of *T. gondii* after treatment with those with best IC_50_ value or good performance in the in silico analysis or first reported for anti-*T. gondii* activity was analyzed by TEM and immunofluorescence microscopy (Figure 2, Figure 3, Figure 4, Figure 5, Figure 6, Figure 7 and Figure 8).

TEM analysis of the untreated control showed a parasitophorous vacuole (PV) with parasites presenting normal morphology and ultrastructural organization (Figure 2A,B). It is possible to observe structures of the apical complex rhoptries (Rp), conoid (C), and micronemes (m). The nucleus (N), dense granules (DG), apicoplast (A), acidocalcisome (Ac), Golgi complex (GC), lipid body (Lb), vacuolar compartment (V), and mitochondria (M) are also evident (Figure 2A,B). Tachyzoites showed a typical division process by endodyogeny (Figure 2B), with the nucleus presenting a horseshoe shape involved by constructing two new daughter cells delimited by the inner membrane complex (IMC; arrows).

Tachyzoites treated with 62.5 nM cycloheximide showed an increased endoplasmic reticulum area (stars) and alterations on the parasite plasma membrane structure, as evidenced by the presence of regions with only a single pellicle (arrowhead) instead of the three-membrane structure composed by the plasmalemma and IMC (Figure 2C). When tachyzoites were treated with 125 nM cycloheximide, it was observed that vacuoles containing parasites were completely lysed; the asterisk evidences a disrupted parasite and its content spread inside the PV (Figure 2D).

Treatment with 62.5 nM bortezomib affected the parasite cell division, with parasites presenting a nucleus with altered morphology, as evidenced by the enclosure of the Golgi complex by the nucleus (arrow in Figure 2E). The arrestment of the division process was also evidenced as single parasites presenting multiple nucleus profiles without the construction of new daughter cells were observed (Figure 2F). Treatment with bortezomib also caused mitochondrial swelling (M in Figure 2E) and affected the pellicle of the parasite, where it is possible to observe regions devoid of the IMC coverage (arrows in Figure 2F).

To confirm the effect of bortezomib on the parasite cell division, we analyzed tachyzoites treated with 31.2 and 62.5 nM for 24 h after labeling with IMC1 and DAPI by immunofluorescence microscopy (Figure 3A–D). Untreated cells (Figure 3A) showed typical morphology (arrow 3) and division process (arrowheads), where it is possible to observe the construction of two new daughter cells delimited by the IMC, with each containing a divided nucleus (arrowheads in Figure 3A). The effect of bortezomib on cell division was observed even when parasites were treated with 31.2 and 62.5 nM (Figure 3B–D). Treated parasites showed large cells with mitotic nuclei without the construction of new daughter cells (arrows). Areas without IMC cover (asterisks) and daughter cells without nuclei (arrowheads) were also observed (Figure 3B–D). Quantification analysis showed that the effect on parasite division is significantly concentration-dependent, as 23.6% and 48.0% of the PVs had parasites with aberrant division after treatment with 31.2 and 62.5 nM, respectively (Figure 3D).

The tachyzoites treated with 100 nM (-)-anisomycin for 48 h showed alteration of the ER architecture (Figure 4A,B). Figure 4A,B show that the ER has a disorganized architecture extending through a large cytoplasmic area. The treatment with 100 nM (-)-anisomycin also arrested parasite division and the plasma membrane structure (Figure 4B), as seen by a PV containing a single large tachyzoite with two divided nuclei (N) without constructing new daughter cells and the lack of IMC at portions of the plasma membrane (arrows), respectively (Figure 4B).

Treatment with 1 μM ivermectin (Figure 4C,D) induced the formation of myelin-like structures [49,50] (arrowhead in Figure 4C and inset), resembling a process of cell death by autophagy. In Figure 4D, tachyzoites show large vacuoles containing membranous material (asterisks) and a lobular nuclear shape. In one parasite, it is possible to observe the construction of two daughter cells (arrowheads in Figure 4D), but a nucleus lobule (arrow) is not involved by one of the daughter cells.

The effects of (-)-anisomycin and Ivermectin on tachyzoite cell division were analyzed with immunofluorescence microscopy after labeling with IMC1 and DAPI (Figure 5A–D). Treatment with 62.5 nM (-)-anisomycin significantly increased the number of PVs containing parasites with aberrant division (22.5%; Figure 5C). The arrow in Figure 5A points to a mass of tachyzoite containing four non-budded daughter cells. The effect of 125 nM (-)-anisomycin was more drastic (Figure 5B,C), leading to the complete arrestment of cell division with PVs containing a sizeable round mass of cells with a nucleus of increased size and with disorganized profiles of IMC through the cytoplasm (arrowheads). As seen by TEM (Figure 4B), parasites presenting regions lacking IMC cover (arrow in Figure 5B) were also observed. Parasites treated with 1 µM ivermectin also presented significant cell division arrestment (Figure 5D,E), with PVs presenting tachyzoites containing a divided nucleus (arrows in Figure 5D) without the formation of new daughter cells.

Treatment of the tachyzoites with 1 µM almitrine also induced myelin-like structures (Figure 6A and inset) and impairment of the parasite’s cell division (Figure 6B). Figure 6B shows the mass of the mother cell with a large vacuole (asterisks) containing two non-budded daughter cells. Large vacuoles with membranous material were also observed (asterisks in Figure 6B). Treatment with 250 µM midostaurin caused drastic effects on the parasite ultrastructure (Figure 6C,D). Parasite masses with daughter cells and IMC profiles spread in the cytoplasm (arrowheads) were observed (Figure 6C). Parasites presenting alterations suggestive of the cell death process, such as the fragmented nucleus (large arrow in Figure 6C), structures resembling the autophagy process (asterisk in Figure 6C), and large masses of parasites with a disrupted cell division process (arrowheads) and in an advanced vacuolization process (asterisks in Figure 6D) were also observed.

The effect on cell division after treatment with almitrine was confirmed after analysis of treated parasites labeled for the IMC and DNA (arrow in Figure 7A). Quantification analysis showed that 23.9% of the vacuoles contained tachyzoites with cell division alteration (Figure 7C). Treatment with Midostaurin was even more drastic (Figure 7B), as more than 80% of vacuoles had aberrant parasites (Figure 7D). Large tachyzoites containing increased-size nuclei (asterisks), regions of the parasite body without IMC cover (arrows), or daughter cells without a nucleus (arrowheads) were observed (Figure 7B).

Treatment with 1.5 μM merimepodib induced Golgi complex fragmentation (GC in Figure 8A), as the stacked membranes were absent and replaced by numerous vesicular structures. Treatment also caused alteration in rhoptry organization and morphology (Rp in Figure 8A and inset). Tachyzoites presenting large vacuoles containing membranous material were also observed after treatment with merimepodib (asterisks in Figure 8B). To better characterize the effect of merimepodib on the rhoptry morphology, parasites treated with 1.5 µM for 24 h were labeled with the antibody against the rhoptry protein ARO, and fluorescence images of six–ten different focal planes (Z) were acquired and analyzed (Figure 8C,D). While untreated parasites showed vacuoles with parasites harboring rhoptries with typical morphology, around 25% of PVs (Figure 8D) showed parasites with rhoptry alteration (arrowheads in Figure 8C) after treatment with merimepodib.

**Figure 9:**
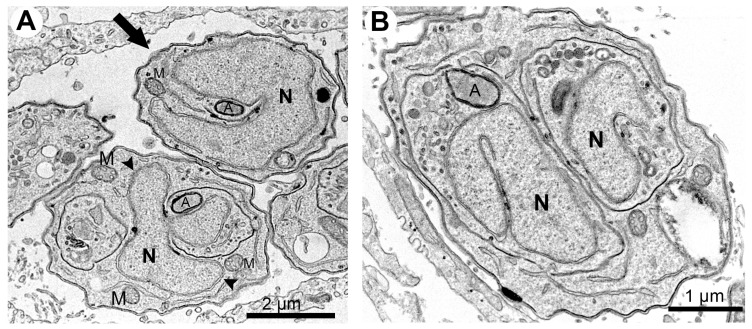
Transmission electron microscopy of *T. gondii* tachyzoites after treatment with 250 nM mycophenolic acid for 48 h. (**A**) Tachyzoites in the division process present multiple lobules (arrowheads) or mitotic nuclei (horseshoe shape) without the construction of new daughter cells (arrow). (**B**) Daughter cells without the completion of the division process with mitotic nuclei. A—apicoplast; M—mitochondria; N—nucleus.

**Figure 10:**
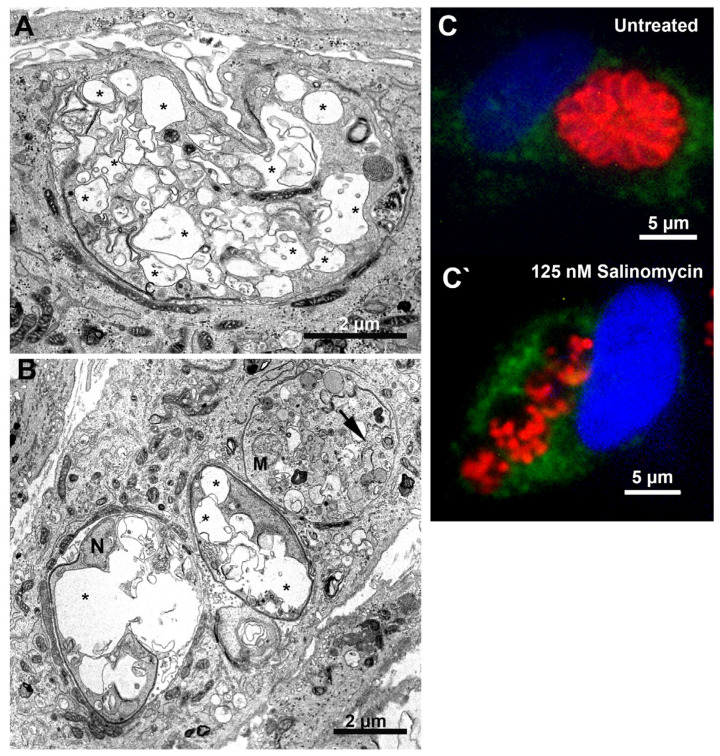
Morphological analysis of *T. gondii* tachyzoites after treatment with salinomycin for 24 h. (**A**) Treatment with 125 nM caused an extensive vacuolization process (asterisks) on the parasite. (**B**) Tachyzoites treated with 250 nM showed an extensive vacuolization process (asterisks) and cell lysis (arrow). (**C**,**C’**) Fluorescence microscopy analysis of tachyzoites treated with 1.5 µM merimepodib for 24 h. Parasites were labeled with anti-LAMP1 for host cell lysosomes (green), anti-SAG1 for parasite plasma membrane (red), and DAPI for DNA (blue).

Treatment with 250 nM mycophenolic acid affected the tachyzoites’ division process (Figure 9A,B). Parasites in the division process presenting multiple lobules (arrowheads in Figure 9A) or mitotic nuclei (horseshoe shape) without the construction of new daughter cells were observed (arrow in Figure 9A). Even daughter cells presented a mitotic nucleus before completing the division process (N in Figure 9B). Tachyzoites treated with 125 and 250 nM salinomycin showed an extensive vacuolization process (asterisks), suggesting an advanced cell death process (Figure 10A,B, respectively). PVs containing lysed parasites (arrow in Figure 10B) were also observed. Analysis by fluorescence microscopy after treatment with 125 nM salinomycin and labeling the parasite plasma membrane (red) and host cell lysosomes (green) showed that differently from untreated cells that had PVs with parasites’ typical morphology and organized in rosettes (Figure 10C), treated parasites showed vacuoles containing fragmented parasites (Figure 10C′). PVs with lysed parasites did not show fusion with lysosomes.

## 4. Discussion

In vitro tests were performed to evaluate the potential of applying the drugs and compounds present in the COVID-Box against *T. gondii* infection and to demonstrate the possibility of the antiproliferative effect of the drugs and compounds in the COVID-Box. After these, we selected 23 drugs and compounds that could inhibit the proliferation of the parasite by more than 70%. The discovery of new uses of drugs previously used for other pharmaceutical purposes is a cheaper solution for treating neglected diseases. The antiproliferative analysis showed that the selected compounds inhibited *T. gondii* proliferation with values of IC_50_s ranging from 0.02 μM to 0.74 μM, and ten showed IC_50_ lower than 100 nM (Table 1). Cytotoxicity analysis by the MTS assay also showed that most compounds were highly selective for *T. gondii* (Table 1). Of the 23 drugs, 11 were recently reported in a study of the anti-*T. gondii* effect of COVID-Box compounds, but two (apilimod and midostaurin) have been reported here for the first time. However, this is the first work to demonstrate the ultrastructural alterations caused by cycloheximide, bortezomib, (-)-anisomycin, ivermectin, almitrine, merimepodib, midostaurin, and salinomycin in tachyzoites through TEM analysis.

Cycloheximide, (-)-anisomycin, and bortezomib are the most potent drugs against *T. gondii* tachyzoites contained in the COVID-Box, inhibiting parasite proliferation with IC_50_s in the range of 20–30 nM. This finding is in line with the study by Fichera, Bhopale, and Ross (1995) [43], which found an IC_50_ value of 0.01 μM for (-)-anisomycin against *T. gondii* after 48 h of treatment. In addition, in silico analyses demonstrated that cycloheximide and (-)-anisomycin show the predictors of good oral bioavailability according to Lipinski’s rule of five (RO5) and Veber, and the potential to cross the BBB (Table 2 and Table S2 and Figure S6). In silico analysis also showed that cycloheximide and (-)-anisomycin are non-P-gp substrates. Published in vivo pharmacokinetic studies (Table S3) showed that cycloheximide and (-)-anisomycin have gastrointestinal absorption and reach plasma concentrations higher than the IC_50_ obtained for *T. gondii*. The brain availability for (-)-anisomycin was also confirmed in vivo. Bortezomib violated only one predictor (Table 2) and showed a prediction for high intestinal permeability (Table S2). A published pharmacokinetic study in humans showed that bortezomib is absorbed and reaches a plasma concentration of 147.6 nM, which is higher than the IC_50_ found for *T. gondii* (Table S3).

Cycloheximide and (-)-anisomycin are bacterial antibiotics isolated from *Streptomyces* species [50]. Although cycloheximide and (-)-anisomycin are protein synthesis inhibitors in eukaryotes, acting during the protein elongation state, their targets on the ribosomes are different. Cycloheximide acts by binding to the E-site and inhibits the mRNA–tRNA translocation. (-)-Anisomycin binds to the A-site, which inhibits the protein synthesis by impairing peptide bond formation, preventing the elongation [50,51,52,53,54,55]. MET analyses showed an increase in the endoplasmic reticulum area of *T. gondii* after treatment with 62.5 nM cycloheximide (Figure 2C) and 100 nM (-)-anisomycin (Figure 4A,B). The endoplasmic reticulum synthesizes essential lipids to maintain the plasma membrane [56,57]. Interestingly, similar ultrastructural changes were observed when *T. gondii* was treated with the antifungal drugs itraconazole, eberconazole, and thiolactomycin analogs [21,29]. The treatment of *T. gondii* with this last drug affected the acylglycerol synthesis by the endoplasmic reticulum [58]. TEM analysis also showed that (-)-anisomycin affected *T. gondii* endodyogeny, confirmed by immunofluorescence microscopy analysis (Figure 5A–C). Interestingly, treatment with 62.5 nM (-)-anisomycin affected the division causing the tethered daughter cell phenotype (Figure 5A), which is typically caused by inhibitors that target the apicoplast pathways, including inhibitors that target the organelle protein synthesis [28,57,58,59]. Treatment with 125 nM (-)-anisomycin disrupted completely parasite division and daughter cell construction (Figure 6B). The increase in the (-)-anisomycin concentration possibly affected other targets, as besides protein synthesis, this drug is also known to inhibit DNA synthesis [60] and to activate stress-activated protein kinases and mitogen-activated protein kinases [50].

A previous study involving bortezomib in tachyzoites of *T. gondii* found an IC_50_ value of 0.10 μM after 72 h of treatment [34]. Cajazeiro et al. (2022) [24] found a different EC_50_ value of 0.22 μM after 72 h of treatment, which differs slightly from the IC_50_ value found in this study after treatment with bortezomib (0.03 μM). This difference is possible due to the longer treatment time used by us in this study, which suggests a time-dependent effect of this drug. Bortezomib is a known potent, selective, and reversible inhibitor of the proteasome, an organelle responsible for the degradation of defective proteins in the cell and crucial for the stability of regulatory proteins. Proteasome inhibitors are known to cause cell death [61,62]. Other authors have reported the inhibition of catalytic subunits of the proteasome in *Plasmodium falciparum* [63,64]. TEM and immunofluorescence microscopy analysis showed that the treatment with 62.5 nM bortezomib significantly affected the parasite division process (Figure 2E,F). As we observed in *T. gondii*, a study that evaluated the effect of bortezomib in a mantle cell lymphoma cell line showed that this drug affected the cell cycle through the G2/M phase arrest [65]. This result is in line with ours, as we also observed parasites with a large undivided nucleus, typical of arrestment of the G2/M phase during the cell division cycle. Therefore, the sum of the results obtained here supports that cycloheximide, (-)-anisomycin, and bortezomib can be potential drugs for treating the acute phase of toxoplasmosis.

This is the first work to study the effect of midostaurin against *T. gondii.* This drug is a potent inhibitor of protein kinase C and several class III receptor tyrosine kinases involved in hematopoiesis and leukemia. It was approved for leukemia treatment and has an oral bioavailability estimated at >90% [66]. Midostaurin showed anti-*T. gondii* IC50 of 80 nM, which is fifteen times lower than its plasma concentration in humans.

TEM and immunofluorescence analysis showed that around 80% of the vacuoles treated with 250 nM of this drug had drastic morphological alterations, such as an aberrant division process and the induction of cell death (Figure 6 and Figure 7). A similar effect was observed in HMC1 cells (neoplastic human mast cells) after treatment with 500 nM midostaurin for 24 h [67].

Ivermectin was another drug with anti-*T. gondii* activity and good pharmacokinetic prediction (Table 1, Table 2 and Tables S2 and S3). The potential activity of ivermectin against *T. gondii* and other protozoa, such as *Giardia lamblia*, *Trypanosoma cruzi*, *Leishmania infantum*, and *Trypanosoma evansi*, has also been reported in the literature [37,68,69,70,71]. However, this is the first study exploring the mode of action of this drug in a parasite from the Apicomplexa phylum. Using TEM, we observed that the treatment with 1 µM ivermectin for 48 h caused multi-membrane structures and large cytoplasmic vacuoles containing membranous material (Figure 4C,D), suggesting induction of cell death by autophagy [72]. Ivermectin also significantly affected the parasite division (Figure 5D,E). A similar effect was observed after treating glioma cells with ivermectin [73]. Treatment of *T. gondii* tachyzoites with almitrine also caused an intense vacuolization and formation of multi-membrane structures (Figure 6A,B) and aberrant cell division (Figure 7A,C), which are suggestive of the induction of an autophagic process [72]. Almitrine is already used in clinics to treat diseases that affect the respiratory system and is a good predictor of oral and brain bioavailability (Table 2 and Table S2). Pharmacokinetic studies on humans showed that it could reach plasma concentrations up to 599 nM (Table S3), which is higher than the IC_50_ obtained for *T. gondii* in this study. The in vitro and in vivo effects against *T. gondii* have been recently reported [23,24], with IC_50_ values of 0.42 μM and 0.32 μM after 72 h of treatment, which are close to what we found in this study. The in vivo administration reduced the number of cysts in a murine model of chronic toxoplasmosis.

Mycophenolic acid and merimepodib are antiviral inhibitors of inositol monophosphate dehydrogenase, affecting DNA and RNA synthesis [74]. These drugs affected *T. gondii* proliferation with IC_50_ of 0.07 µM and 0.48 µM, respectively. Previous studies showed IC_50_s of 211 µM (mycophenolic acid) and 0.78 µM (merimepodib) for *T. gondii* tachyzoites after 24 and 72 h of treatment, respectively [23]. Merimepodib also showed a high selective index for *T. gondii* compared with HFF cells (human foreskin fibroblasts) [23]. Analyses by TEM showed that merimepodib caused *T. gondii* Golgi complex fragmentation, rhoptry disorganization, and intense vacuolation (Figure 8A,B). The effect against rhoptries was analyzed by immunofluorescence, confirming that this is a significant alteration on the parasite (Figure 8C). Similar results were observed in tachyzoites after depletion of the vacuolar protein sorting nine (*Tg*Vps9), Vps11, and a membrane inositol phospholipid binding protein [75,76,77]. These results suggest that this drug could affect *T. gondii*, interfering with its secretory pathway. Although merimepodib and mycophenolic acid target the same enzyme, tachyzoites treated with the latter showed a different mode of action, as cell division alteration was the main observed effect after treatment with mycophenolic acid. A previous study also showed rounded tachyzoites with multiple nucleus profiles after treatment with mycophenolic acid [39]. This drug is also widely used in studies of molecular manipulation of *T. gondii* for selecting mutants that express the selectable marker HXGPRT [78].

We also investigated the mode of action of the H^+^/K^+^ ionophore salinomycin on *T. gondii*, a known anticoccidial drug commonly used for poultry and cattle [79,80]. TEM analysis showed that salinomycin induces parasite death, causing its lysis. Immunofluorescence analysis confirmed that cell death is directly caused by this drug and not by a secondary effect due to the fusion of lysosomes with the PVs.

Developing a new infectious disease treatment is complex because medicines must be absorbed, reach adequate plasma concentrations, and be distributed to tissues and cellular compartments where the infection is present in the body. In the case of toxoplasmosis, this is even more critical, as one of the main sites of infection is the CNS. Based on an anti-*T. gondii* activity assay and according to Lipinski’s and Veber’s predictors’ analysis, the 23 drugs and compounds identified in this work are good candidates to become oral drugs since they inhibit *T. gondii* proliferation at a submicromolar range and comply with RO5, showing no more than one violation. We can highlight that the compounds cycloheximide, bortezomib, anisomycin, almitrine, midostaurin, and mycophenolic acid presented an IC_50_ range lower than 0.10 μM and did not violate Lipinski’s rules and the predictors of Veber. Ivermectin and merimepodib are good candidates, inhibiting *T. gondii* with an IC_50_ < 0.5 µM. In addition, these drugs also demonstrated desirable predictors for oral absorption (Table 2 and Table S2 and S3). However, we should not disregard the potential of drugs that did not comply with RO5 or presented values lower than expected for oral absorption or BBB permeability since AZT, a drug already commercialized, presented a value lower than expected (−0.211 log Papp at 10^−6^ cm/s) and intestinal absorption (human = 45.808%), but despite AZT not having good intestinal absorption, this drug is used to treat toxoplasmosis [9].

## 5. Conclusions

After COVID-Box screening, we identified two new drugs with anti-*T. gondii* activity, making this the first study to report their effectiveness against this parasite. In total, 23 drugs were found to be promising candidates for further pre-clinical studies on toxoplasmosis. The discovery of these new drug candidates for the treatment of toxoplasmosis is of great relevance and should be further explored for in vivo analysis in the future. The results presented here have shown that drug repurposing is a potential alternative for treating infectious and neglected diseases and that the boxes provided by MMV are crucial for solving the problems involved in treating these diseases.

## Figures and Tables

**Table 1:** IC_50_ values and cytotoxicity of drugs and compounds of COVID-Box.

Plate Position	Trivial Name	Disease Area	Target	Status ^a^	IC_50_ (μM) in Tachyzoites	Cytotoxicity for NHDF		Anti-*T. gondii* Activity ^c^
						CC_50_ (μM)	SI ^b^	
AA02	Niclosamide	Antiparasitic	Anaerobic phosphorylation inhibitor	Approved	0.36 ± 0.02	3.05 ± 0.08	8	[23,35]
AA04	Bemcentinib	Immune agent	Tyrosine kinase	Phase II	0.15 ± 0.03	0.97 ± 0.15	6	[23]
AB03	Apilimod	Antitumor	PIKfyve inhibitor	Phase II	0.22 ± 0.06	2.12 ± 0.12	10	New
AB04	Regorafenib	Antitumor	Tyrosine kinase	Approved	0.25 ± 0.04	3.03 ± 0.01	12	[23]
AC10	LY2228820	Antitumor agent	p38 mitogen-activated protein kinase inhibitor	Phase II, discontinued	0.04 ± 0.00	nd	nd	[23]
AD02	Digoxin	Antiarrhythmic	Na^+^/K^+^-ATPase inhibitor	Approved	0.03 ± 0.01	0.34 ± 0.32	11	[24]
AE06	Emetine	Antiparasitic	Unknown	Approved	0.05 ± 0.01	1.02 ± 0.19	20	[36]
AF05	Ivermectin	Antiparasitic	Agonist of glutamate-gated Cl^−^channels	Approved	0.21 ± 0.01	nd	nd	[37]
AF09	Sorafenib	Antitumor	Tyrosine kinase inhibitor	Approved	0.56 ± 0.03	nd	nd	[23]
AG03	Manidipine	Antihypertensive	Calcium channel blocker	Phase III	0.74 ± 0.09	nd	nd	[23]
AG04	Almitrine	Respiratory system	Mitochondrial ATP synthase	Approved *	0.33 ± 0.04	nd	n.d	[23,24]
AG08	Midostaurin	Antitumor	Tyrosine kinase	Approved	0.08 ± 0.00	0.24 ± 0.02	3	New
AG11	Abemaciclib	Antitumor	Cyclin-dependent kinase	Approved	0.09 ± 0.01	#	#	[23]
AH03	Tetrandrine	Antitumor	P-glycoprotein	Preclinical	0.35 ± 0.07	4.77 ± 8.25	14	[23]
BA07	Ponatinib	Antitumor	Tyrosine kinase	Approved	0.33 ± 0.03	4.84 ± 0.89	15	[34]
BA09	Berbamine	Antitumor	CAMKII inhibitor	Preclinical	0.31 ± 0.03	4.61 ± 7.52	15	[23]
BB10	Mycophenolic acid	Immunosuppressant	Inosine monophosphate dehydrogenase	Approved	0.07 ± 0.01	23.81 ± 31.97	340	[38,39]
BD02	Salinomycin	Anti-microbial agent	Alkali ion carrier	Approved	0.07 ± 0.01	1.14 ± 0.17	16	[40]
BD08	Merimepodib	Antiviral agent	Inosine monophosphate dehydrogenase	Phase II	0.48 ± 0.08	nd	nd	[23]
BD11	Cycloheximide	Agricultural agent– Fungicide	Protein synthesis inhibitor	Research	0.02 ± 0.00	6.08 ± 4.63	304	[41,42]
BF06	(-)-Anisomycin	Anti-infective agent	Protein synthesis	Approved	0.02 ± 0.00	0.25 ± 0.13	13	[43]
BG06	Bortezomib	Antitumor	Proteasome inhibitor	Approved	0.03 ± 0.00	0.72 ± 0.24	24	[24,34]
BG07	Pimozide	Antipsychotic	Dopamine receptor	Approved	0.64 ± 0.15	nd	nd	[24,33]

nd = not determined. Concentrations up to 3 µM did not affect NHDF cell proliferation. # There was not enough to perform this assay. ^a^ Approved: FDA-approved drug; Phase I or II or III: Clinical candidate drug in Phase 1, 2, or 3 clinical trials; * Almitrine was withdrawn in some countries. ^b^ Selectivity index. ^c^ We searched PubMed for the trivial name of each compound plus “*Toxoplasma gondii*”. If available, the reference for a previous study was included. The term “New” indicates when no results were retrieved from the search.

**Table 2:** Physical-chemical properties of drugs and compounds of COVID-Box according to Lipinski’s RO5 and predictors of Veber.

Identification	LogP ^a^	H-Bond Donors	H-Bond Receptors	MW ^b^	Violations Lipinski	TPSA (Å^2^) ^c^	nº Rotations	Violations Veber
Pyrimethamine	2.84	2	4	248.7	0	77.83	2	0
Sulfadiazine	0.86	3	5	250.3	0	98.57	3	0
Clindamycin	0.39	4	7	424.9	0	102.25	7	1
Azithromycin	2.50	5	14	749.0	2	198.54	7	1
Atovaquone	5.34	1	3	366.8	1	54.70	2	0
Niclosamide	3.85	2	4	327.1	0	128.62	3	0
Bemcentinib	4.88	2	8	506.7	2	92.35	4	0
Apilimod	3.08	1	8	418.5	0	84.77	8	0
Regorafenibe	4.39	3	7	482.8	0	92.35	5	0
LY2228820	5.51	2	5	420.5	1	85.41	5	0
Digoxin	2.22	6	14	780.9	3	203.06	7	1
Emetine	3.04	1	6	480.7	0	52.20	7	0
Ivermectin	4.37	3	14	875.1	2	170.06	8	1
Sorafenib	4.10	3	7	464.8	0	92.35	9	0
Manidipine	4.04	1	8	610.7	1	116.94	12	1
Almitrine	4.34	2	6	477.6	0	69.21	10	0
Midostaurin	4.05	1	4	570.6	1	77.73	4	0
Abemaciclib	4.04	1	8	506.6	1	75.00	7	0
Tetrandrine	5.49	0	8	622.7	2	61.86	4	0
Ponatinib	4.30	1	8	532.6	1	65.77	6	0
Berbamine	5.13	1	8	608.7	2	72.86	3	0
Mycophenolic acid	2.72	2	6	320.3	0	93.07	6	0
Salinomycin	4.98	4	11	751.0	2	161.21	12	2
Merimepodib	2.36	3	7	452.5	0	123.96	11	1
Cycloheximide	1.23	2	4	281.4	0	83.47	3	0
Anisomycin	1.00	2	5	265.3	0	67.79	5	0
Bortezomib	0.22	4	6	384.2	0	124.44	11	1
Pimozide	5.67	1	5	461.5	1	41.29	7	0

^a^ LogP: octanol–water partition coefficient; ^b^ MW: molecular weight; ^c^ TPSA: total polar surface area.

## Data Availability

The original contributions presented in this study are included in the paper. Further inquiries can be directed to the corresponding author.

## Supplementary Materials

The following supporting information can be downloaded at https://www.mdpi.com/article/10.3390/microorganisms12122602/s1, Table S1: Plate position and trivial name of each compound of COVID-Box; Table S2: Pharmacokinetic properties of drugs and compounds of COVID-Box according to pkCSM; Table S3: In vivo bioavailability parameters of the best COVID-Box molecules; Figure S1: Preliminary evaluation of the effectiveness of 160 drugs and compounds from COVID-Box against *T. gondii* tachyzoites; Figure S2: Post-treatment recovery assay of *T. gondii* RH strain tachyzoites after treatment with the 23 drugs and compounds of the COVID-Box at a concentration of 1 µM; Figure S3: Chemical structure of 23 drugs and compounds the COVID Box; Figure S4: Antiproliferative effect of 23 drugs and compounds the COVID-Box after seven days treatment. After obtaining the NDHF cell monolayer, the cells were infected with 600 *T. gondii* RH strain tachyzoites; Figure S5: Cell viability of host cells infected with *T. gondii* tachyzoites after treatment with the 23 drugs and compounds selected from the COVID-Box after seven days of incubation; Figure S6: Boiled-egg graph obtained through the SwissADME platform.
