# Supplementary material for: Antiproliferative and Morphological Analysis Triggered by Drugs Contained in the Medicines for Malaria Venture COVID-Box Against Toxoplasma gondii Tachyzoites
Source: Microorganisms. 2024 Dec 16;12(12):2602. doi: 10.3390/microorganisms12122602 (PMC11676817; doi:10.3390/microorganisms12122602)
Supplement: Supplementary file 1 [file microorganisms-12-02602-s001.zip › microorganisms-3180219-supplementary.pdf]

Supplementary material

Supplementary Table S1

Table S1. Plate Position and Trivial Name of each compound of Covid-Box

Plate A			Plate B	
Plate Position	Trivial Name		Plate Position	Trivial Name
AA02	Niclosamide		BA02	Fluspirilene
AA03	Lusutrombopag		BA03	Tetracycline
AA04	Bemcentinib		BA04	Toremifene
AA05	ONO 5334		BA05	Doxorubicin
AA06	Remdesivir		BA06	Rapamycin
AA07	N-Desethylamodiaquine		BA07	Ponatinib
AA08	Ciclesonide		BA08	Migalastat
AA09	Nelfinavir		BA09	Berbamine
AA10	Ethaverine		BA10	Metformin
AA11	Ritonavir		BA11	Fluconazole
AB02	Chlorpromazine		BB02	Lumefantrine
AB03	Apilimod		BB03	Anidulafungin
AB04	Regorafenib		BB04	Hanfangchin B
AB05	Pexidartanib		BB05	Entacapone
AB06	Ketoconazole		BB06	Thiethylperazine
AB07	Drotaverine		BB07	Tacrolimus
AB08	Verapamil		BB08	Dabrafenib
AB09	R 7112		BB09	Dapivirine
AB10	Atazanavir		BB10	Mycophenolic acid
AB11	Ruxolitinib		BB11	Tenofovir
AC02	Papaverine		BC02	Silmitasertib
AC03	GSK-369796		BC03	Oxyclozanide
AC04	Daclatasvir		BC04	Digitoxin
AC05	Lonafarnib		BC05	Chlormidazole
AC06	Itraconazole		BC06	Proscillaridin
AC07	GSK 983		BC07	Dutacatib
AC08	Tioguanine		BC08	PB 28
AC09	Tomivosertib		BC09	Baricitinib
AC10	LY 2228820		BC10	Eszopiclone
AC11	Tigecycline		BC11	(+)-Mefloquine
AD02	Digoxin		BD02	Salinomycin
AD03	Amodiaquine		BD03	JQ 1
AD04	Astemizole		BD04	RVX 208
AD05	Halofantrine		BD05	Osimertinib
AD06	Ravuconazole		BD06	Cloperastine
AD07	Tizoxanide		BD07	Clemastine
AD08	Valproic Acid		BD08	Merimepodib
AD09	Simeprevir		BD09	E 52862
AD10	Favipiravir		BD10	Doravirine
AD11	Vidofludimus		BD11	Cycloheximide

AE02	Hydroxyprogesterone		BE02	Umifenovir
AE03	Nafamostat		BE03	MRT 68601
AE04	Amuvatinib		BE04	PD 144418
AE05	Doxycycline		BE05	(RS)-PPCC
AE06	Emetine		BE06	AZ3451
AE07	Triparanol		BE07	ZINC4326719
AE08	Clomipramine		BE08	Losartan
AE09	Sofosbuvir		BE09	Nifedipine
AE10	Camostat		BE10	Anagliptin
AE11	SMN-C3		BE11	Desmethyl ferroquine
AF02	Valsartan		BF02	Nebivolol
AF03	Amiodarone		BF03	Aprepitant
AF04	Cyclosporine		BF04	Danusertib
AF05	Ivermectin		BF05	Apremilast
AF06	Indomethacin		BF06	(-)-Anisomycin
AF07	Captopril		BF07	Idelalisib
AF08	Ribavirin		BF08	Darifenacin
AF09	Sorafenib		BF09	Spectinomycin
AF10	ABT 239		BF10	Oxatomide
AF11	Hydroxychloroquine		BF11	Promethazine
AG02	Benztropine		BG02	Veliparib
AG03	Manidipine		BG03	Celecoxib
AG04	Almitrine		BG04	Cabozantinib
AG05	Nitazoxanide		BG05	Selumetinib
AG06	MK-2206		BG06	Bortezomib
AG07	Terconazole		BG07	Pimozide
AG08	Midostaurin		BG08	Sertindole
AG09	Ferroquine		BG09	Boceprevir
AG10	SAX-187		BG10	Valdecoxib
AG11	Abemaciclib		BG11	Paroxetine
AH02	Pevonedistat		BH02	Darunavir
AH03	Tetrandrine		BH03	Brequinar
AH04	Lopinavir		BH04	Delanzomib
AH05	Imatinib		BH05	Delavirdine
AH06	Fluphenazine		BH06	Indinavir
AH07	Haloperidol		BH07	Abacavir
AH08	Ozanimod		BH08	Chlorothiazide
AH09	Loratidine		BH09	Apixaban
AH10	Posaconazole		BH10	Naphtoquine
AH11	Pyronaridine		BH11	Piperaquine

Supplemental Table S2

Table S2. Pharmacokinetic properties of drugs and compounds of Covid-Box according to pkCSM.

Identification	Caco-2 Permeability[a]	Intestinal Absorption (human)	Fraction Unbound (human)	VDss (Human)[b]	CNS permeability[c]	BBB permeability[d]
**Pyrimethamine**	0.927	92.74%	0.311	-0.307	-2.203	-0.166
**Sulfadiazine**	0.702	73.92%	0.28	0.182	-2.87	-0.672
**Clindamycin**	0.063	53.28%	0.747	-0.206	-3.63	-0.943
**Azithromycin**	-0.211	45.81%	0.719	-0.214	-4.12	-1.494
**Atovaquone**	1.483	91.41%	0	0.329	-1.418	0.401
**Niclosamide**	0.886	89.48%	0	-0.022	-1.972	-0.626
**Bemcentinib**	1.654	91.04%	0.152	0.799	-2.097	-0,953
**Apilimod**	1.388	96.75%	0.044	-0.173	-2.901	-1.047
**Regorafenibe**	0.454	93.43%	0	-0.277	-2.031	-1.573
**LY2228820**	1.321	84.39%	0.367	0.066	-1.674	-1.378
**Digoxin**	0.381	78.23%	0.287	0.085	-4.19	-1.927
**Emetine**	1.333	95.40%	0.155	1.632	-2.041	0.045
**Ivermectin**	0.602	89.45%	0.126	0.587	-3.438	-2.000
**Sorafenib**	0.907	84.99%	0	-0.105	-1.995	-1.675
**Manidipine**	0.928	93.59%	0.086	0.683	-2.339	-0.998
**Almitrine**	1.392	87.59%	0.136	1.189	-2.727	-0.945
**Midostaurin**	0.985	98.28%	0.286	-1.663	-1.764	0.039
**Abemaciclib**	1.341	89.68%	0.097	0.614	-2.981	-1.665
**Tetrandrine**	0.737	92.83%	0.371	-0.808	-2.576	0.074
**Ponatinib**	1.000	91.44%	0.040	0.565	-1.862	0.278
**Berbamine**	1.143	92.79%	0.399	-0.999	-2.608	-0.936
**Mycophenolic acid**	0.244	62.24%	0.217	-0.651	-2.908	-0.159
**Salinomycin**	-0.140	58.65%	0.220	0.372	-3.068	-1.754
**Merimepodib**	1.024	93.76%	0	-0.122	-3.32	-1.647
**Cycloheximide**	0.467	69.78%	0.51	-0.042	-2.996	-0.162
**(-) -Anisomycin**	0.244	80.65%	0.574	0.134	-2.932	-0.301
**Bortezomib**	0.292	54.25%	0.235	-0.736	-4.102	-1.397
**Pimozide**	0.399	85.97%	0.137	0.071	-1.314	0.101

[a]log Papp in 10^{-6} cm/s; [b]Volume of distribution (log L/kg) - low if below -0.15; high if above 0.45; [c]compounds with logPS > -2 are predicted to penetrate CNS, and with logPS < -3 unable to penetrate; [d]compounds with logBB > 0.03 are considered to readily cross BBB and < -1 poorly permeable.

Supplemental Table S3

Table S3. ***In vivo*** **bioavailability parameters of the best Covid-box molecules**

**Drug**	**Plasma concentration (C_{max}[1])**		**Does it cross the blood-brain barrier or is found in the brain?**	
Abemaciclib	Human	492 ng/mL (971 nM) [1]	Mouse and Rat	Yes [2]
(-) -Anisomycin	Rat	2.75 ng/mL (10 nM) [3]	Rat	Yes [3]
Almitrine	Human	286.2 ng/mL (599 nM) [4]	-	N.D.[2]
Apilimod	Mice	2.53 μM [5]	Mice	Yes [6]
	Human	1h – 225 ng/mL (537.6 nM)<br>6h – 50 ng/mL (119.5 nM) [7]	-	N.D.
Bemcentinib	Human	53 ng/mL (104.6 nM) [8]	Human	Yes [9]
Berbamine	Rat	32.6 ng/mL (53.6 nM) [10]	-	ND
Bortezomib	Human	56.7 ng/mL (147.6 nM) [11]	-	N.D.
Cycloheximide	Rat	43.1 ng/mL (153.2 nM) [12]	-	N.D.
Emetine	Human	9.6 ng/mL (20 nM) [13]	Human	Yes[14]
Ivermectin	Human	260.5 ng/mL (297.7 nM) [15]	Mice	Yes [16]
	Mouse	642.3 ng/mL (733.9 nM) [16]		
Manidipine	Human	5.53 ng/mL (9 nM) [17]	-	N.D.
Merimepodib	Human	1590 ng/mL (5514 nM) [18]	-	N.D.
Midostaurin	Human	1210 ng/mL (2120.6 nM) [19]	-	N.D.
Mycophenolic acid	Human	24.3 mg/L (75.8 μM) [20]	-	N.D.
Niclosamide	Rat	354 ng/mL (1.08 μM) [21]	-	N.D.
	Human	1.28 μg/mL (3.9 μM) [22]	-	N.D.
Ponatinib	Human	123 ng/mL (231 nM) ([23]	Mouse	Yes [24]
Regorafenib	Human	1.9 mg/L (3.9 μM) [25]	Human	Yes [26]
Salinomycin	Mice	13.2 μg/L (17.6 nM) [27]	Mouse	Yes [27]
	Rat	40 μg/L (53.3 nM) [28]	Rat	N.D.
Sorafenib	Rat	2.41 μg/mL (5.2 μM) [29]	Rat and Monkey	Yes [30]
	Human	1001.3 ng/mL (2.2 μM) [31]	-	ND
Tetrandrine	Rat	237.10 μg/L (381 nM) [32]	Mice	Yes [33]

[1] maximum observed drug concentration; [2]N.D. No data found

References

1. Raub, T.J.; Wishart, G.N.; Kulanthaivel, P.; Staton, B.A.; Ajamie, R.T.; Sawada, G.A.; Gelbert, L.M.; Shannon, H.E.; Sanchez-Martinez, C.; De Dios, A. Brain Exposure of Two Selective Dual CDK4 and CDK6 Inhibitors and the Antitumor Activity of CDK4 and CDK6 Inhibition in Combination with Temozolomide in an Intracranial Glioblastoma Xenograft. *Drug Metab Dispos* **2015**, *43*, 1360–1371, doi:10.1124/dmd.114.062745.
2. Groenland, S.L.; Martínez-Chávez, A.; Van Dongen, M.G.J.; Beijnen, J.H.; Schinkel, A.H.; Huitema, A.D.R.; Steeghs, N. Clinical Pharmacokinetics and Pharmacodynamics of the Cyclin-Dependent Kinase 4 and 6 Inhibitors Palbociclib, Ribociclib, and Abemaciclib. *Clin Pharmacokinet* **2020**, *59*, 1501–1520, doi:10.1007/s40262-020-00930-x.
3. Tolić, L.; Grujić, S.; Mojović, M.; Jovanović, M.; Lubec, G.; Bačić, G.; Laušević, M. Determination of Anisomycin in Tissues and Serum by LC-MS/MS: Application to Pharmacokinetic and Distribution Studies in Rats. *RSC Adv.* **2016**, *6*, 92479–92489, doi:10.1039/C6RA16083B.
4. Stavchansky, S.; Doluisio, J.T.; MacLeod, C.M.; Szalkowski, M.B.; Bachand, R.T.; Heilman, R.; Sebree, T.B.; Geary, R.S. Single Oral Dose Proportionality Pharmacokinetics of Almitrine Bismesylate in Humans. *Biopharm & Drug Disp* **1989**, *10*, 229–237, doi:10.1002/bdd.2510100302.
5. Nelson, E.A.; Dyall, J.; Hoenen, T.; Barnes, A.B.; Zhou, H.; Liang, J.Y.; Michelotti, J.; Dewey, W.H.; DeWald, L.E.; Bennett, R.S.; et al. The Phosphatidylinositol-3-Phosphate 5-Kinase Inhibitor Apilimod Blocks Filoviral Entry and Infection. *PLoS Negl Trop Dis* **2017**, *11*, e0005540, doi:10.1371/journal.pntd.0005540.
6. Guerrero-Valero, M.; Grandi, F.; Cipriani, S.; Alberizzi, V.; Di Guardo, R.; Chicanne, G.; Sawade, L.; Bianchi, F.; Del Carro, U.; De Curtis, I.; et al. Dysregulation of Myelin Synthesis and Actomyosin Function Underlies Aberrant Myelin in CMT4B1 Neuropathy. *Proc. Natl. Acad. Sci. U.S.A.* **2021**, *118*, e2009469118, doi:10.1073/pnas.2009469118.
7. Harb, W.A. Phase 1 Clinical Safety, Pharmacokinetics (PK), and Activity of Apilimod Dimesylate (LAM-002A), a First-in-Class Inhibitor of Phosphatidylinositol-3-Phosphate 5-Kinase (PIKfyve), in Patients with Relapsed or Refractory B-Cell Malignancies.
8. Bhalla, S.; Fattah, F.J.; Ahn, C.; Williams, J.; Macchiaroli, A.; Padro, J.; Pogue, M.; Dowell, J.E.; Putnam, W.C.; McCracken, N.; et al. Phase 1 Trial of Bemcentinib (BGB324), a First-in-Class, Selective AXL Inhibitor, with Docetaxel in Patients with Previously Treated Advanced Non-Small Cell Lung Cancer. *Lung Cancer* **2023**, *182*, 107291, doi:10.1016/j.lungcan.2023.107291.
9. Burt Nabors, L.; Nakano, I.; Supko, J.; Lobbous, M.; Grossman, S.; Ye, X.; Desideri, S.; Danda, N.; Fisher, J.; Strowd, R.; et al. CTNI-07. ABTC-1701: PILOT SURGICAL PK STUDY OF BGB324 (BEMCENTINIB) IN RECURRENT GLIOBLASTOMA PATIENTS – RESULTS FROM INTERIM FUTILITY ANALYSIS. *Neuro-Oncology* **2021**, *23*, vi60–vi60, doi:10.1093/neuonc/noab196.232.
10. Feng, X.; Wang, K.; Cao, S.; Ding, L.; Qiu, F. Pharmacokinetics and Excretion of Berberine and Its Nine Metabolites in Rats. *Front. Pharmacol.* **2021**, *11*, 594852, doi:10.3389/fphar.2020.594852.
11. Reece, D.E.; Sullivan, D.; Lonial, S.; Mohrbacher, A.F.; Chatta, G.; Shustik, C.; Burris, H.; Venkatakrishnan, K.; Neuwirth, R.; Riordan, W.J.; et al. Pharmacokinetic and Pharmacodynamic Study of Two Doses of Bortezomib in Patients with Relapsed Multiple Myeloma. *Cancer Chemother Pharmacol* **2011**, *67*, 57–67, doi:10.1007/s00280-010-1283-3.
12. Al Nebaihi, H.M.; Davies, N.M.; Brocks, D.R. Pharmacokinetics of Cycloheximide in Rats and Evaluation of Its Effect as a Blocker of Intestinal Lymph Formation. *European Journal of Pharmaceutics and Biopharmaceutics* **2023**, *193*, 89–95, doi:10.1016/j.ejpb.2023.10.016.
13. Scharman, E.J.; Hutzler, J.M.; Rosencrance, J.G.; Tracy, T.S. Single Dose Pharmacokinetics of Syrup of Ipecac: *Therapeutic Drug Monitoring* **2000**, *22*, 566–573, doi:10.1097/00007691-200010000-00011.
14. Parmer, Leo G. et al. Distribution of Emetine in Tissues. *The Journal of Laboratory and Clinical Medicine* **1949**, *34*, 818–821.
15. Guzzo, C.A.; Furtek, C.I.; Porras, A.G.; Chen, C.; Tipping, R.; Clineschmidt, C.M.; Sciberras, D.G.; Hsieh, J.Y.; Lasseter, K.C. Safety, Tolerability, and Pharmacokinetics of Escalating High Doses of Ivermectin in Healthy Adult Subjects. *The Journal of Clinical Pharma* **2002**, *42*, 1122–1133, doi:10.1177/009127002237994.
16. Geyer, J.; Gavrilova, O.; Petzinger, E. Brain Penetration of Ivermectin and Selamectin in *Mdr1a,b* P-glycoprotein- and *Bcrp* - Deficient Knockout Mice. *Vet Pharm & Therapeutics* **2009**, *32*, 87–96, doi:10.1111/j.1365-2885.2008.01007.x.

17. Jing, J.; Ren, W.; Chen, X.; He, H.; Zhou, W.; Zhu, X.; Sun, Y.; Wang, G. Determination and Pharmacokinetics of Manidipine in Human Plasma by HPLC/ESIMS. *Biomedical Chromatography* **2007**, *21*, 836–840, doi:10.1002/bmc.827.
18. Rustgi, V.K.; Lee, W.M.; Lawitz, E.; Gordon, S.C.; Afdhal, N.; Poordad, F.; Bonkovsky, H.L.; Bengtsson, L.; Chandorkar, G.; Harding, M.; et al. Merimepodib, Pegylated Interferon, and Ribavirin in Genotype 1 Chronic Hepatitis C Pegylated Interferon and Ribavirin Nonresponders. *Hepatology* **2009**, *50*, 1719–1726, doi:10.1002/hep.23204.
19. He, H.; Tran, P.; Gu, H.; Tedesco, V.; Zhang, J.; Lin, W.; Gatlik, E.; Klein, K.; Heimbach, T. Midostaurin, a Novel Protein Kinase Inhibitor for the Treatment of Acute Myelogenous Leukemia: Insights from Human Absorption, Metabolism, and Excretion Studies of a BDDCS II Drug. *Drug Metab Dispos* **2017**, *45*, 540–555, doi:10.1124/dmd.116.072744.
20. Bullingham, R.E.S.; Nicholls, A.J.; Kamm, B.R. Clinical Pharmacokinetics of Mycophenolate Mofetil: *Clinical Pharmacokinetics* **1998**, *34*, 429–455, doi:10.2165/00003088-199834060-00002.
21. Chang, Y.-W.; Yeh, T.-K.; Lin, K.-T.; Chen, W.-C.; Yao, H.-T.; Lan, S.-J.; Wu, Y.-S.; Hsieh, H.-P.; Chen, C.-M.; Chen, C.-T. Pharmacokinetics of Anti-SARS-CoV Agent Niclosamide and Its Analogs in Rats. *Journal of Food and Drug Analysis* **2020**, *14*, doi:10.38212/2224-6614.2464.
22. Walther, N.; Schultz-Heienbrok, R.; Staß, H.; Corman, V.M.; Gassen, N.C.; Müller, M.A.; Drosten, C.; Witzenrath, M.; Lee, H.; Posch, M.G. Clinical Safety and Pharmacokinetics of a Novel Oral Niclosamide Formulation Compared with Marketed Niclosamide Chewing Tablets in Healthy Volunteers: A Three-Part Randomized, Double-Blind, Placebo-Controlled Trial 2024.
23. Hanley, M.J.; Diderichsen, P.M.; Narasimhan, N.; Srivastava, S.; Gupta, N.; Venkatakrishnan, K. Population Pharmacokinetics of Ponatinib in Healthy Adult Volunteers and Patients With Hematologic Malignancies and Model-Informed Dose Selection for Pediatric Development. *The Journal of Clinical Pharma* **2022**, *62*, 555–567, doi:10.1002/jcph.1990.
24. Ravi, K.; Franson, A.; Homan, M.J.; Roberts, H.; Pai, M.P.; Miklja, Z.; He, M.; Wen, B.; Benitez, L.L.; Perissinotti, A.J.; et al. Comparative Pharmacokinetic Analysis of the Blood-Brain Barrier Penetration of Dasatinib and Ponatinib in Mice. *Leukemia & Lymphoma* **2021**, *62*, 1990–1994, doi:10.1080/10428194.2021.1894647.
25. Weekes, C.; Lockhart, A.C.; Lee, J.J.; Sturm, I.; Cleton, A.; Huang, F.; Lenz, H. A Phase 1b Study Evaluating the Safety and Pharmacokinetics of Regorafenib in Combination with Cetuximab in Patients with Advanced Solid Tumors. *Intl Journal of Cancer* **2019**, *145*, 2450–2458, doi:10.1002/ijc.32317.
26. Zeiner, P.S.; Kinzig, M.; Divé, I.; Maurer, G.D.; Filipski, K.; Harter, P.N.; Senft, C.; Bähr, O.; Hattingen, E.; Steinbach, J.P.; et al. Regorafenib CSF Penetration, Efficacy, and MRI Patterns in Recurrent Malignant Glioma Patients. *JCM* **2019**, *8*, 2031, doi:10.3390/jcm8122031.
27. Lagas, J.S.; Sparidans, R.W.; Van Waterschoot, R.A.B.; Wagenaar, E.; Beijnen, J.H.; Schinkel, A.H. P-Glycoprotein Limits Oral Availability, Brain Penetration, and Toxicity of an Anionic Drug, the Antibiotic Salinomycin. *Antimicrob Agents Chemother* **2008**, *52*, 1034–1039, doi:10.1128/AAC.01041-07.
28. Resham, K.; Patel, P.N.; Thummuri, D.; Guntuku, L.; Shah, V.; Bambal, R.B.; Naidu, V.G.M. Preclinical Drug Metabolism and Pharmacokinetics of Salinomycin, a Potential Candidate for Targeting Human Cancer Stem Cells. *Chemico-Biological Interactions* **2015**, *240*, 146–152, doi:10.1016/j.cbi.2015.08.007.
29. Karbownik, A.; Stanisławiak-Rudowicz, J.; Stachowiak, A.; Romański, M.; Grześkowiak, E.; Szałek, E. The Influence of Paracetamol on the Penetration of Sorafenib and Sorafenib N-Oxide Through the Blood–Brain Barrier in Rats. *Eur J Drug Metab Pharmacokinet* **2020**, *45*, 801–808, doi:10.1007/s13318-020-00639-z.
30. Kim, A.; McCully, C.; Cruz, R.; Cole, D.E.; Fox, E.; Balis, F.M.; Widemann, B.C. The Plasma and Cerebrospinal Fluid Pharmacokinetics of Sorafenib after Intravenous Administration in Non-Human Primates. *Invest New Drugs* **2012**, *30*, 524–528, doi:10.1007/s10637-010-9585-1.
31. Díaz-González, Á.; Sapena, V.; Boix, L.; Brunet, M.; Torres, F.; LLarch, N.; Samper, E.; Millán, O.; Corominas, J.; Iserte, G.; et al. Pharmacokinetics and Pharmacogenetics of Sorafenib in Patients with Hepatocellular Carcinoma: Implications for Combination Trials. *Liver International* **2020**, *40*, 2476–2488, doi:10.1111/liv.14587.
32. Song, N.; Zhang, S.; Li, Q.; Liu, C. Establishment of a Liquid Chromatographic/Mass Spectrometry Method for Quantification of Tetrandrine in Rat Plasma and Its Application to Pharmacokinetic Study. *Journal of Pharmaceutical and Biomedical Analysis* **2008**, *48*, 974–979, doi:10.1016/j.jpba.2008.06.002.
33. Tong, B.C.-K.; Wu, A.J.; Huang, A.S.; Dong, R.; Malampati, S.; Iyaswamy, A.; Krishnamoorthi, S.; Sreenivasmurthy, S.G.; Zhu, Z.; Su, C.; et al. Lysosomal TPCN (Two Pore Segment Channel) Inhibition Ameliorates Beta-Amyloid Pathology and Mitigates Memory Impairment in Alzheimer Disease. *Autophagy* **2022**, *18*, 624–642, doi:10.1080/15548627.2021.1945220.

Supplemental Figure S1

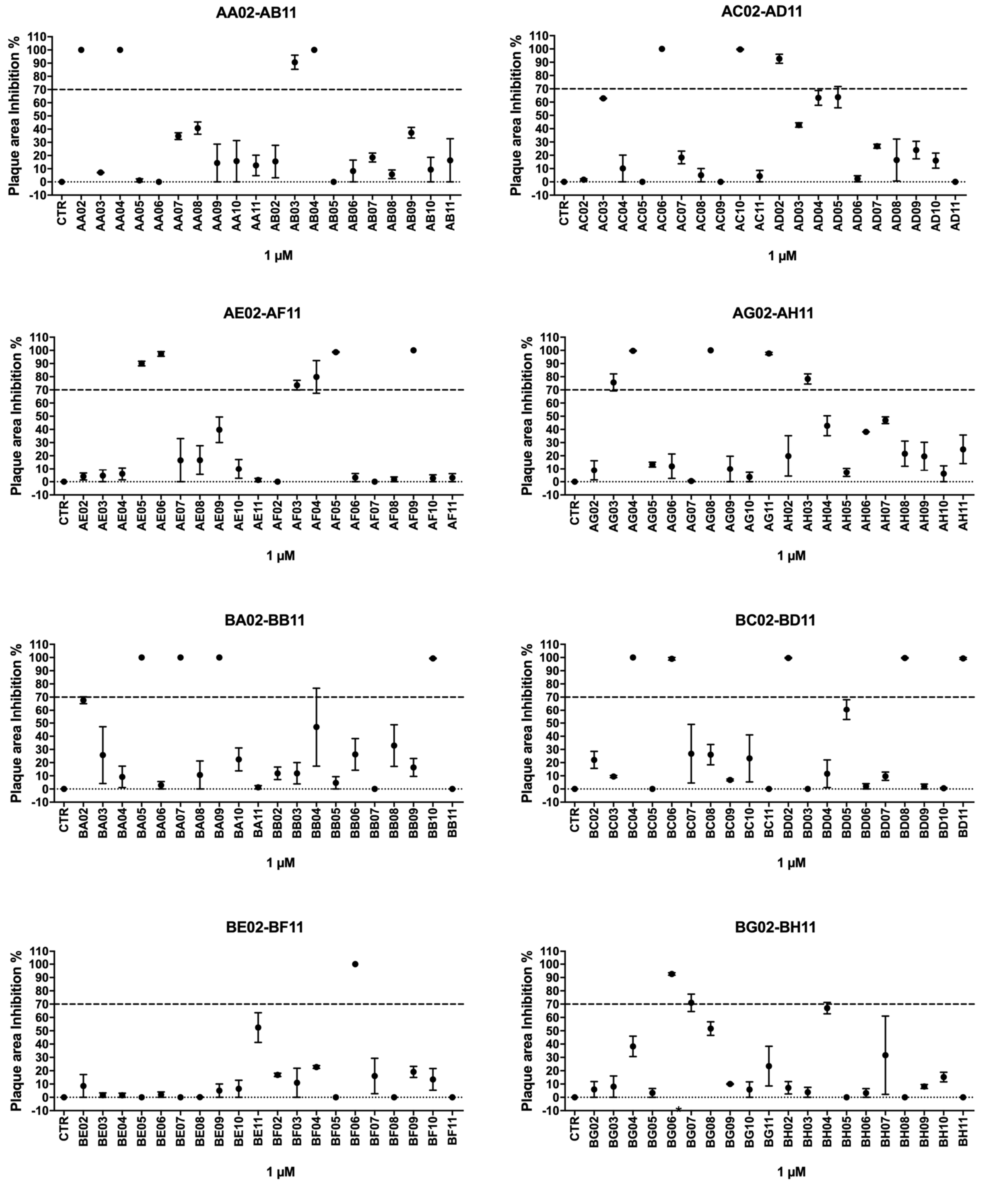

Figure S1. Preliminary evaluation of the effectiveness of 160 drugs and compounds from Covid-Box against *T. gondii* tachyzoites. After obtaining the NDHF cell monolayer, the cells were infected with 1,000 RH strain tachyzoites. After infection, each well of the plate was treated at a concentration of 1µM of each drug. Values represent the mean ± SD of two experiments. CTR= Control.

Supplemental Figure S2

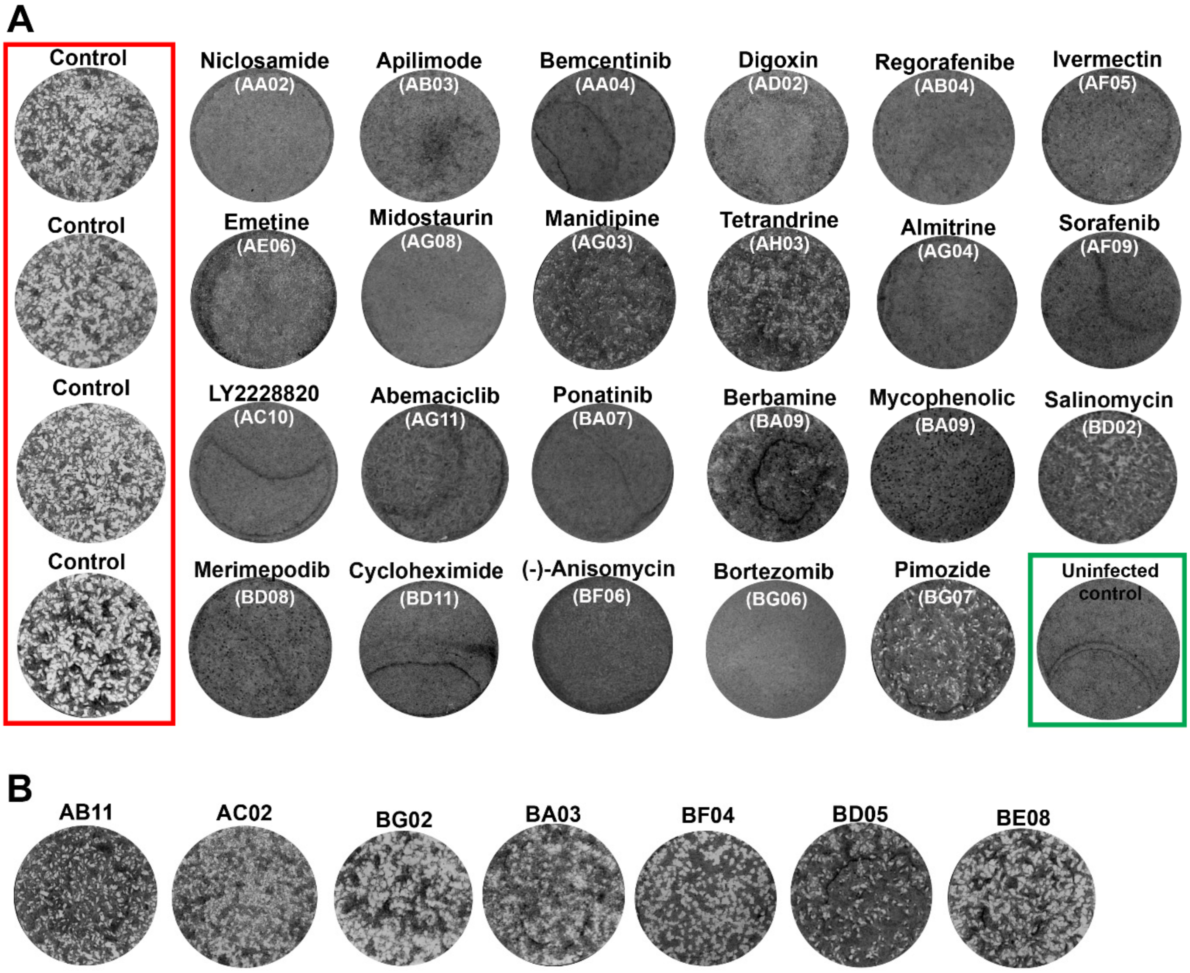

Figure S2. Plaque assay images after treatment with the drugs and compounds of the Covid Box at a concentration of 1 µM. (A) Wells of the 23 Covid-Box drugs and compounds that showed proliferation inhibition above 70%. (B) Wells of some compounds that inhibited proliferation below 70%. Red rectangle: infected control wells from different plates. Green rectangle: Vehicle control well (uninfected cells).

Supplemental Figure S3

A

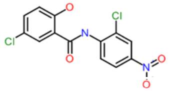

1. (AA02)

Niclosamide

B

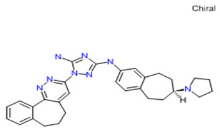

2. (AA04)

Bemcentinib

C

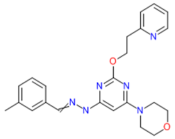

3. (AB03)

Apilimod

D

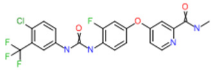

4. (AB04)

Regorafenib

E

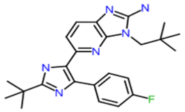

5. (AC10)

LY2228820

F

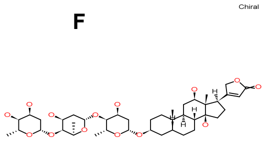

6. (AD02)

Digoxin

G

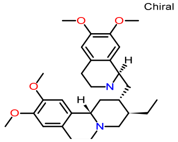

7. (AE06)

Emetine

H

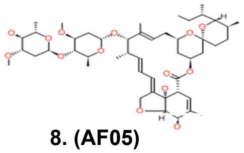

8. (AF05)

Ivermectin

I

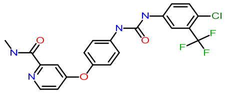

9. (AF09)

Sorafenib

J

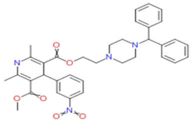

10. (AG03)

Manidipine

K

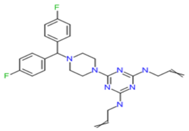

11. (AG04)

Almitrine

L

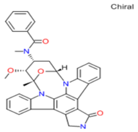

12. (AG08)

Midostaurin

M

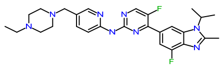

13. (AG11)

Abemaciclib

N

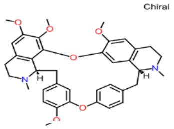

14. (AH03)

Tetrandrine

O

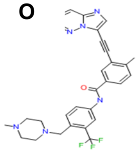

15. (BA07)

Ponatinib

P

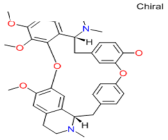

16. (BA09)

Berbamine

Q

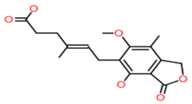

17. (BB10)

Mycophenolic acid

R

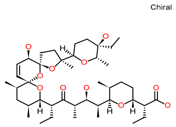

18. (BD02)

Salinomycin

S

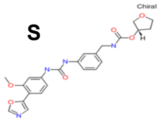

19. (BD08)

Merimepodib

T

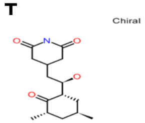

20. (BD11)

Cycloheximide

U

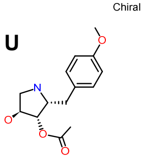

21. (BF06)

V

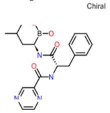

22. (BG06)

Bortezomib

W

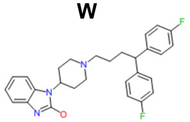

23. (BG07)

Pimozide

Figure S3. Chemical structure of 23 drugs and compounds the Covid Box. (MMV, 2023).

Supplemental Figure S4

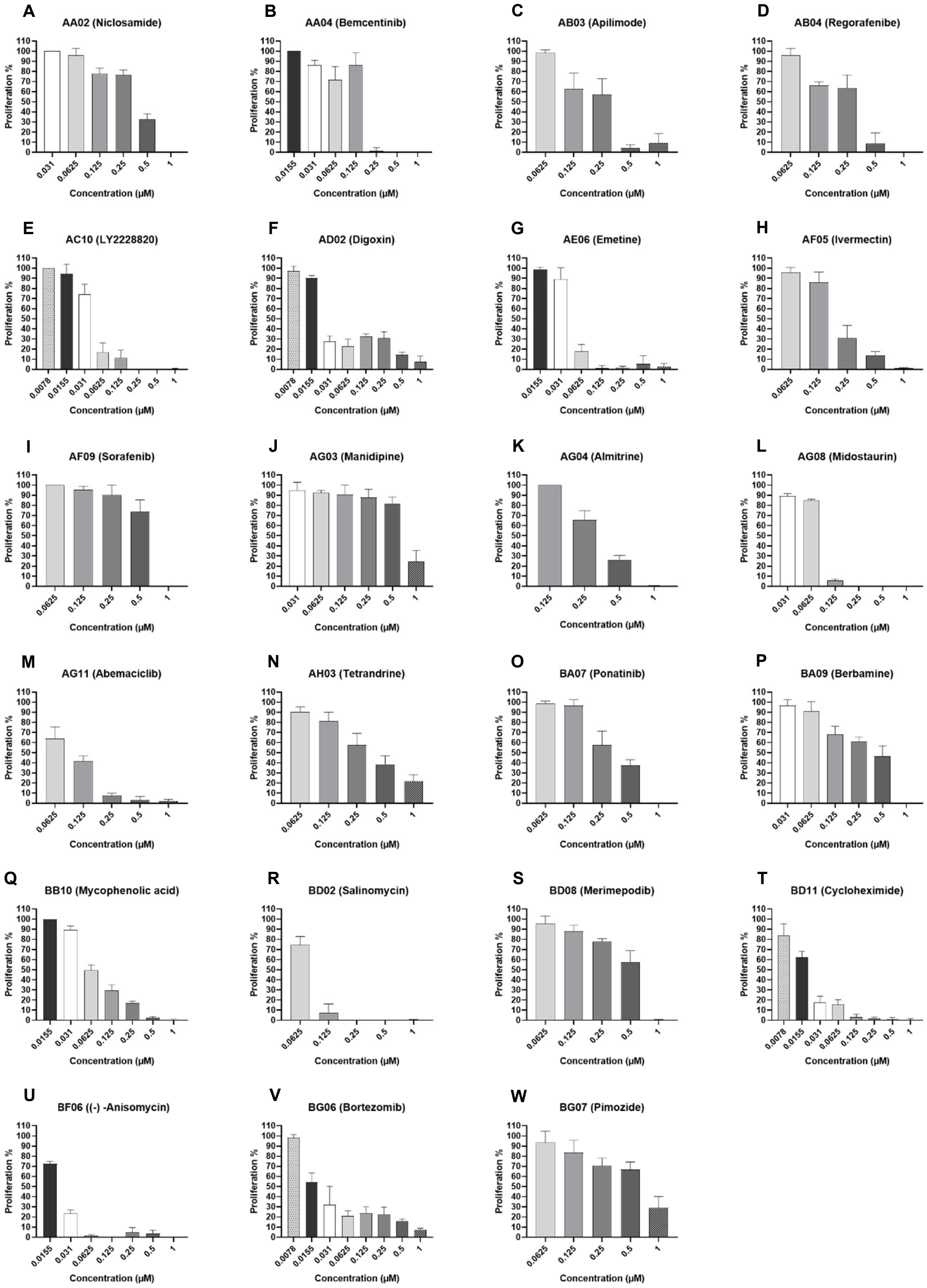

Figure S4. The antiproliferative effect after treating tachyzoite-infected NHDF monolayers with different concentrations of the 23 drugs and compounds. Cells were infected with 600 *T. gondii* RH strain tachyzoites and treated for 7 days. Values represent the mean ± SD of three experiments. CTR = Control.

Supplemental Figure S5

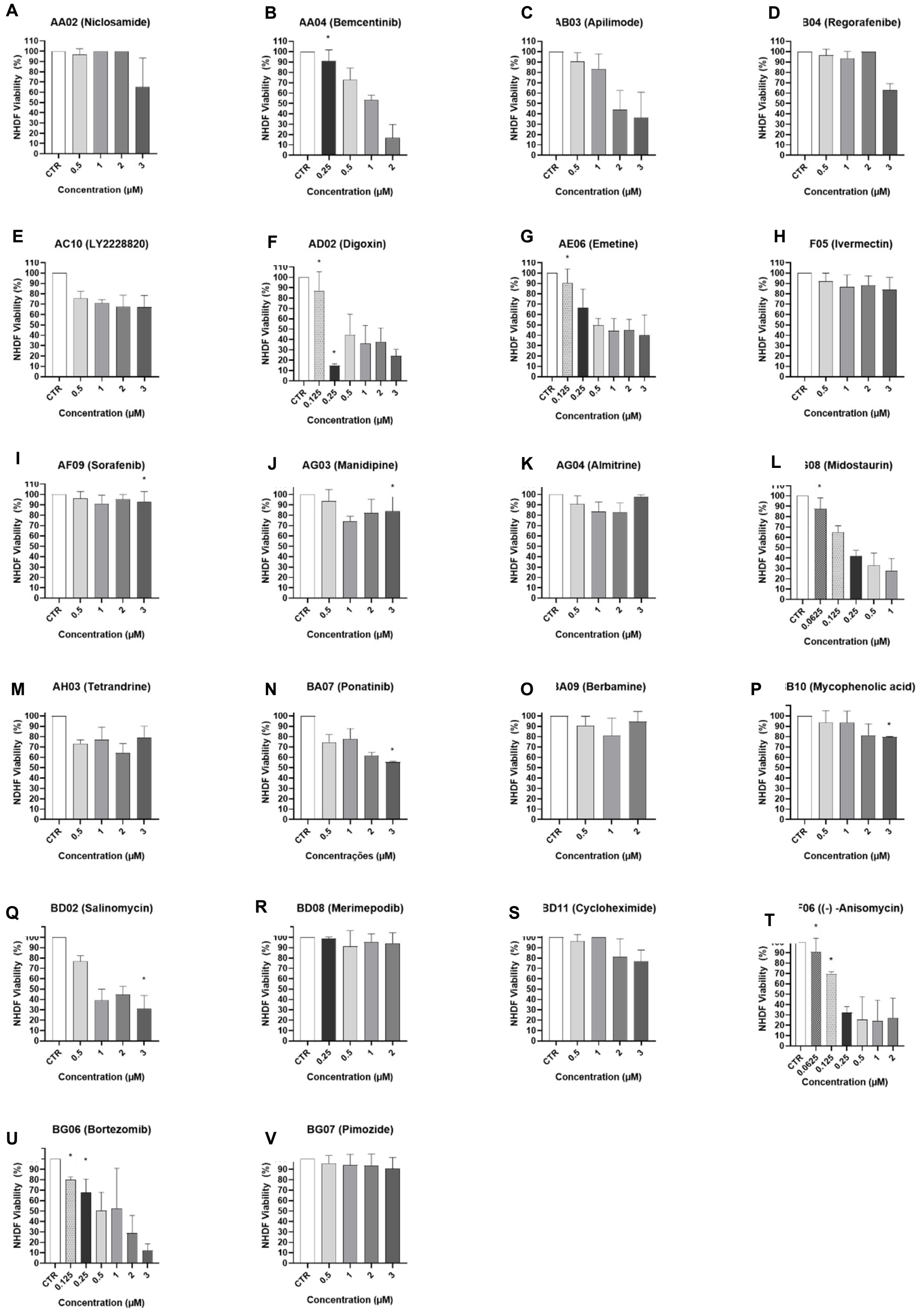

Figure S5. Viability of NHDF cells after a 7-day treatment with different concentrations of the 23 drugs and compounds. Values represent the mean ± SD of three experiments. *Indicates only two independent experiments at this concentration. CTR= Control.

Supplemental Figure S6

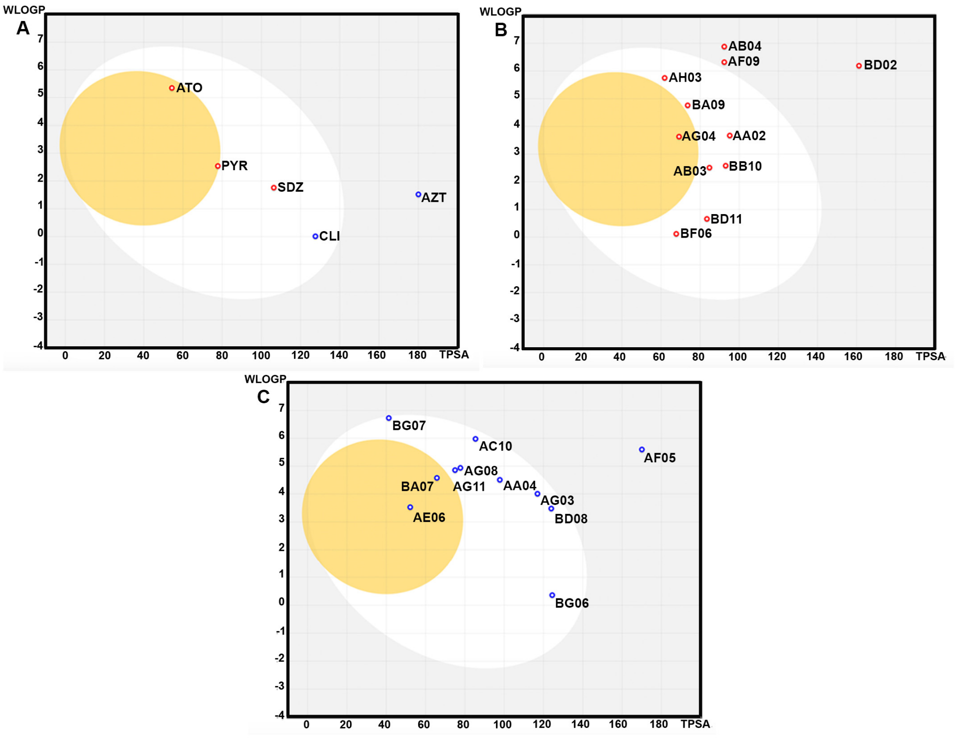

Figure S6. A-C. Boiled-Egg graph obtained through the SwissADME platform. The yellow part represents LogBB values = compounds that show highly probable blood-brain barrier (BBB) permeation; the white part represents HIA values = highly probable gastrointestinal absorption; Red dots represent PGP- = non-P-gp substrate; Blue dots represent PGP+ = P-gp substrate. (A) PYR= Pyrimethamine; SDZ = Sulfadiazine; CLI = Clindamycin; AZT = Azithromycin; ATO = Atovaquone. (B) AA02 = Niclosamide; AB03 = Apilimod; AB04 = Regonofarib; AF09 = Sorafenib; AG04 = Almitrine; AH03 = Tetrandrine; BA09 = Berbamine; BB10 = Mycophenolic acid; BD02 = Salinomycin; BD11 = Cycloheximide; BF06 = (-)-Anisomycin. (C) AA04 = Bemcentinib; AC10= LY2228820; AD02= Digoxin (out of coverage area); AE06= Emetine; AF05 = Ivermectin; AG03 = Manidipine; AG08 = Midostaurin; AG11= Abemaciclib; BA07= Ponatinib; BD08 = Merimepodib; BG06= Bortezomib; BG07 = Pimozide.
